# Role of intestinal microbiota and metabolites on gut homeostasis and human diseases

**DOI:** 10.1186/s12865-016-0187-3

**Published:** 2017-01-06

**Authors:** Lan Lin, Jianqiong Zhang

**Affiliations:** 1 Department of Bioengineering, Medical School, Southeast University, Nanjing, 210009 People’s Republic of China; 2 Key Laboratory of Developmental Genes and Human Disease, Ministry of Education, Department of Microbiology and Immunology, Medical School, Southeast University, Nanjing, 210009 People’s Republic of China

**Keywords:** Intestinal microbiota, Gut homeostasis, Immune responses, Regulatory T cells (Tregs), Dendritic cells (DCs), Metabolic disorder

## Abstract

**Background:**

A vast diversity of microbes colonizes in the human gastrointestinal tract, referred to intestinal microbiota. Microbiota and products thereof are indispensable for shaping the development and function of host innate immune system, thereby exerting multifaceted impacts in gut health.

**Methods:**

This paper reviews the effects on immunity of gut microbe-derived nucleic acids, and gut microbial metabolites, as well as the involvement of commensals in the gut homeostasis. We focus on the recent findings with an intention to illuminate the mechanisms by which the microbiota and products thereof are interacting with host immunity, as well as to scrutinize imbalanced gut microbiota (dysbiosis) which lead to autoimmune disorders including inflammatory bowel disease (IBD), Type 1 diabetes (T1D) and systemic immune syndromes such as rheumatoid arthritis (RA).

**Results:**

In addition to their well-recognized benefits in the gut such as occupation of ecological niches and competition with pathogens, commensal bacteria have been shown to strengthen the gut barrier and to exert immunomodulatory actions within the gut and beyond. It has been realized that impaired intestinal microbiota not only contribute to gut diseases but also are inextricably linked to metabolic disorders and even brain dysfunction.

**Conclusions:**

A better understanding of the mutual interactions of the microbiota and host immune system, would shed light on our endeavors of disease prevention and broaden the path to our discovery of immune intervention targets for disease treatment.

## Background

Human gastrointestinal tract is known to host trillions of microbes [1, 2], the number of which reaches approximately 10^14^ cells in the entire gut of a healthy individual [1]. Amongst these resident gut microbes, 4000 strains are present constituting the intestinal microbiota [3]. Through co-evolution, the host has not only tolerated but also evolved to necessitate the colonization by beneficial microbes, termed commensals, for multifaceted aspects of immune development and function [4]. Defects in mucosal tolerance are believed to cause human disorders including inflammatory bowel disease (IBD) exemplified by Crohn’s disease and ulcerative colitis [5].

As the first line defense of host against pathogens, innate immune responses rely on a family of receptors known as pattern recognition receptors (PRRs) including Toll-like receptors (TLRs), and nucleotide-binding oligomerization domain-like (NOD-like) receptors. TLRs are key innate immune receptors to perceive pathogen-associated molecular patterns (PAMPs), which are specific pathogenic “molecular signature” [6]. Subsequent to sensing microbial PAMPs, TLRs enable the initiation of inflammatory responses and eventually eliminate the pathogenic invaders. The phenomenon that both commensals and pathogenic microbes can interact with host immune system through similar conserved ligands —PAMPs, drives us to address such question as to how host immune system differentiates pathogens from commensals at the intestinal mucosal interface exposed to continuous microbial stimuli.

Severe host tissue damage may be resulted from immune hypersensitivity towards intestinal flora or dietary nutrients. To circumvent this, the host implements a variety of regulatory mechanisms for organ homeostasis maintenance. Regulatory T cells (Tregs) serve one such mechanism as evidenced by the otherwise catastrophic consequences under genetic/or physical ablation of the Treg population [7]. Tregs are the specialized T cells with immunosuppressive activity through an array of mechanisms that influence both dendritic cells (DCs) and effector cells [8].

DCs, constituting the first point of contact between gut commensals and mammalian immune system [9], are central to harmonizing the host tolerance (to self-antigens) with host immunity (to pathogens) in the peripheral lymphoid tissues [10]. DCs are able to present innocuous self and non-self antigens in a manner that promotes tolerance [8]. The predominant mechanism by which DCs induce and maintain peripheral tolerance involves the generation of Tregs from naïve T cells, the expansion of pre-existing Tregs, the production of IL-10 and other immunomodulatory cytokines, and the promotion of T cell anergy or depletion [11, 12].

Immature DCs (iDCs), present in all peripheral tissues, are capable of acquiring antigenic material from their microenvironment, but are poorly immunogenic (also called tolergenic). The pathogenic microbial signals can be sensed by iDCs for propelling their conversion into mature DCs, which, present within secondary lymphoid organs, could obtain the capacity of promoting T cell immunity but lose the capacity of antigen uptake [13]. In short, DCs are able to trigger seemingly opposite states —— immunity and tolerance depending on different microenvironment conditions [13]. Intestinal DCs, together with macrophages and epithelial cells, may serve as sentinels in the microbial milieu of intestine. The exceptional characteristic of intestinal microenvironment necessitates host immune system not only to avoid the hyper-immune reactivity to the gut lumen laden with commensals and dietary components etc, but also to retain the capacity of fighting pathogenic microbes.

Extensive studies in germ-free (GF) mice, in the past decades, have demonstrated an indispensable role of microbiota in shaping host intestine immune system [14]. In contrast to conventionally raised mice, GF mice have hypoplastic Peyer’s patches, decreased numbers in IgA-secreting plasma cells and lamina propria CD4+ T cells, relatively structureless secondary lymphoid tissues (i.e. spleen and peripheral lymph nodes) and other immunologic defects. Inoculation of a healthy murine commensal microbiota into GF mice has been found to reverse these immunologic deficiencies [14]. In addition to immunostimulatory effects as afore-described, certain members of intestine microbiota may exert immunomodulatory actions that involve reversible alterations in differentiation/or effector function of host immune cell subsets, exemplified by segmented filamentous bacteria (SFB), *Bacteroides fragilis*, Clostridia XIVa and IV. This aspect will be reviewed in details in the Section of “Commensals and gut homeostasis”. Furthermore, compelling evidence with microbiota-derived metabolites, mainly referring to small-molecule constituents such as short-chain fatty acids (SCFAs) and quorum sensing signals, has established the importance of chemical signaling in communicating microbial richness and composition with host. And microbial metabolites can be sensed by host immune system in addition to PAMPs, which in turn influences host immune responses. Butyrate, a kind of microbiota-originated SCFAs containing four carbons, has been recently reported to have immunomodulatory effects on intestinal macrophages and thereby conferring them hyporesponsive to commensal microbiota residing in the colon [15]. Notwithstanding*,* the underlying mechanisms as to how intestinal microbiota, as a whole, educates host immune system within the gut and beyond, as well as the identification of bacterial species-specific contribution during the microbiota-host immunity interaction still await to be elucidated. As a paradigm of bacterial strain-specific molecules, butyrate acts as HDAC inhibitors and ligands for G-protein-coupled receptors (GPCRs) and is considered as a crucial signaling molecule affecting host immune responses [16].

Majority of human lymphoid tissue is located within the lining of the major tracts that are predominant entry sites of microbes into host, referring to respiratory, gastrointestinal (GI) and genitourinary tracts, which are collectively termed the mucosa-associated lymphoid tissues. The intestinal mucosa appears to be the largest surface within human body facing enormous amounts of microbial antigens either resident or ingested. This review summarizes the recent advances in the field of microbiota and their products interacting with the GI mucosal immune system. We aim to provide an update into the research progress relevant to the possible contributions of microbiota and their products to the intestinal homeostasis maintenance, which, hopefully, would facilitate the virtual discovery and insightful design of promising therapeutic targets for treatment of human disorders in association with intestinal dysbiosis and autoimmunity, such as type 1 diabetes (T1D), systemic immune syndromes (i.e. IBD etc.) and even colorectal cancer.

## Review

### Effects of gut microbe-derived nucleic acids on immunity

#### TLR9 senses unmethylated cytidine-phosphate-guanosine (CpG) motifs of DNA

Host cells can initiate innate immune signaling upon recognition of PAMPs (viz. conserved structures in pathogenic microbes), of which nucleic acids are key structures. The receptors for foreign nucleic acids involve members of TLRs including TLR3, TLR7, TLR8, and TLR9, and intracellular DNA sensors [17]. The endosomal localizations of TLR3 [activated by double-stranded (ds) RNA], TLR7 and 8 [activated by single-stranded (ss) RNA], TLR9 [activated by CpG motifs within ssDNA] reflect the protective mechanism whereby unwanted interactions of TLRs with self-nucleic acids could be circumvented. Another protective mechanism may involve modifications of mammalian nucleic acids [18]. Detection of intracellular pathogens is achieved by those endosomally-expressed TLR3, and TLRs 7–9, eventually leading to the clearance of pathogens.

Among those TLRs in association with intracellular invaders, TLR9 and signaling thereof are more extensively investigated than others. Unmethylated CpG dinucleotides that are enriched in prokaryotic DNAs of intestinal flora, can be sensed by TLR9. Constitutive gut flora DNA sensing is found to modulate the equilibrium between regulatory and effector T cells in the murine GI tract, suggesting the gut flora DNA as an immunological adjuvant [19]. Moreover, unmethylated CpG has been reported of immunostimulatory effect in mice and other mammals, as well as in-vitro human cell lines [20, 21]. Bacterial DNA and synthetic oligonucleotiodes (ODN), which contain unmethylated CpG in common, are able to activate the innate and adaptive immune system *via* plasmacytoid dendritic cells (pDCs) and macrophages in mammals [22].

Upon CpG stimulation, a signaling cascade is elicited that leads to the production of proinflammatory cytokines and type I IFNs [23, 24], the latter being predominantly secreted by pDC. These soluble components coordinate early innate and sequential adaptive immune responses [24]. The tissue specificity and cellular pattern of TLR expression are believed to vary with different species, even in mammals. For instance, murine TLR9 is expressed not only in pDC and B cells as human TLR9, but also in macrophages and myeloid DCs as well [21]. Thus one should be cautious with predicting the effects of TLR9 activation on humans by extrapolating from murine data.

#### TLR9 signaling and autoimmunity

Several lines of evidence have revealed inappropriate activations of TLR7, TLR8, and TLR9 in systemic lupus erythematosus (SLE) and several other autoimmune diseases. T and B cells specific for self-antigens can be detected in healthy individuals but do not suffice to provoke the development of autoimmune diseases. In contrast, SLE individuals are reported to suffer from impaired clearance of apoptotic cells and increased circulating levels of nucleosomes [18]. CpG motifs derived from apoptotic debris could activate TLR9, notably under the circumstance that they are converted into immune complexes with pre-existing auto-antibodies, followed by B cells stimulation through both TLR9 and B-cell receptor, which in turn leads to autoimmunity and systemic autoimmune disease [25]. In such SLE individuals, host DNA/antibody complexes trigger and sustain a pDC- and B cell-mediated immune response [26, 27], which indicates self-DNA as damage-associated molecular pattern (DAMP) modulating self-destructive chronic immune activation [28].

Studies have characterized several proteins as intermediate cofactors (chaperones) to initiate the TLR9 activation upon perception of CpG, which include human cathelicidin LL-37 and the high mobility group box (HMGB). Cathelicidin LL-37, a cationic peptide with wide-spectrum antimicrobial activities, is chemotactic for neutrophils, mast cells, monocytes, and T cells [29]. In psoriasis patients LL-37 may serve as a converter of self-DNA into pathogenic ligand due to its binding to self-DNA. The resultant LL37-DNA complex is found to promote the endocytosis pathway and to sustain TLR9 activation by modifying the interaction with DNA [30]. Accordingly, LL37 facilitates TLR9 activation of self-DNA and synthetic CpG DNA. CpG islands under study were demonstrated to be immunostimulatory when coupled with human cathelicidin LL-37, strongly suggesting the critical role of LL-37 in the immunostimulatory effects of CpG motif-containing mtDNA fragments [24].

TLR9 recognizes not only CpG motifs “embedded” in bacterial DNA but also similar motifs in vertebrate DNA, pinpointing that the same receptor perceives PAMP and DAMP, which complies with the notion that the immune system is more concerned with entities that do damage than those that are foreign [31]. It also indicates that similarities exist between pathogen-induced responses and non-infectious inflammatory responses [32]. CpG motifs in prokaryotic DNA are known to be 20 times more enriched than those in mammalian DNA; and even found in the mammalian genomic DNA, they are specifically methylated. MtDNA is predominantly unmethylated in view of its prokaryotic origin based on endosymbiosis theory [33]. Once eukaryotic cells undergo apoptosis, necrosis, necroptosis and cell death in association with autophagy, mtDNA is released acting as mtDAMP. On the other hand, neutrophils, basophils and eosinophils, upon stimulation, can release extracellular traps of mtDNA or genomic DNA. These traps contain such antimicrobial peptides as cathelicidins and cell-specific proteases. A growing body of evidence has revealed that elevated levels of circulating mtDNA may cause systemic inflammatory response syndrome in trauma patients and also act as a trigger of neurodegeneration [34, 35]. The pDC may be stimulated by an influx of neutrophils releasing extracellular traps of DNA [36], and are subsequently recruited to the colorectum and gut mucosa [37, 38]. Accordingly, fragmented mtDNA bearing CpG motif may contribute to driving a Th1 polarization in autoimmune disorder and chronic viral diseases [24].

#### TLR9 signaling and gut cancinoma

CpG-mediated TLR9 activation may serve as a new therapeutic target for several cancerous conditions. The potentials of TLR9 agonists (synthetic CpG ODN) in therapeutic applications for infectious diseases, cancer and asthma/allergy have been reviewed elsewhere [21].

Recent studies have determined the association of TLR9 polymorphisms with human susceptibility to gastric carcinoma and its prognosis in Chinese population [39]. The work by Wang et al strongly suggests that TLR9-1486C carriers are associated with an increased risk and poor prognosis of gastric carcinoma in human [39]. Another independent group has shown the cell-invasion-inducing potential of short DNA sequences and bacterial DNAs in tested cell lines including human MDA-MB-231 breast cancer, OE33 esophageal adenocarcinoma, AGS gastric adenocarcinoma and Caco-2 colon carcinoma [40]. An array of DNA ligands was investigated including short DNA sequences such as CpG-ODN M362, 9-mer (hairpin), human telomeric sequence h-Tel22 G-quadruplex, and bacterial DNAs derived from *Escherichia coli* and *Helicobacter pylori* [40]. DNA-induced invasion was shown to be suppressed by a broad-spectrum matrix metalloproteinase (MMP) inhibitor and in part by chloroquine, suggestive of its mediation through endosomal signaling, TLR9 and MMP activation. This notion is reminiscent of the association of MMP overexpression with breast cancer brain metastasis [41]. The work by Kauppila et al. strongly suggests that bacterial DNAs could act as endogenous and invasion-triggering TLR9 ligands and thereby accelerating local progression and metastasis of carcinoma in the digestive tract [40].

#### Immunmodulatory effects of gut microbiota-derived DNA

It awaits elucidating how commensals communicate with host cells to ensure immune homeostasis. As widely known, commensals contain abundant oligodeoxynucleotides with CpG motifs (CpG-ODN), the latter of which has been shown to co-stimulate T cells analogous to that achieved by CD28 stimulation, irrespective of antigen-presenting cells (APCs). The inherent attribute of CpG-ODN towards T cells may contribute to the adjuvanticity potency of microbital DNA and CpG-ODN on T-cell-mediated immune responses [42].

Recent work with gut commensals demonstrated gut-floral-derived DNA (gfDNA) as an intrinsic adjuvant to prime intestinal immune responses, in which TLR9 signaling is involved [19]. TLR9 signaling was found to lower the activation threshold by negative and positive expansions of Treg and Teff (effector T) cells, respectively, in the gut, and was liable to development of protective responses upon oral infection. Thus gfDNA is strongly suggested to be a natural adjuvant for initiating protective immune responses via modulation of Treg/Teff cell ratio at sites of mucosal challenge, which offers promising therapeutic strategy against oral infection [19].

Another independent work with suppressive DNA motifs of the commensal origin showed that these oligonucleotides could contribute to the hierarchy of commensal-derived signals and thereby facilitating the maintenance of gut immune homeostasis [43]. Commensal DNA was previously demonstrated to promote intestinal immunity. It has been unveiled that the bacterial species-specific immunomodulatory capacity of DNA is correlated with the frequency of motifs exerting immunosuppressive action [43]. For instance, DNAs of *Lactobacillus* species, together with those of various probiotics, are known to be enriched in suppressive motifs capable of inhibiting DC activation within lamina propria of intestine. In addition, immunosuppressive oligonucleotides could sustain Treg cell conversion during inflammation, and regulate pathogen-triggered immunopathology and colitis. Collectively, these data pinpoint the suppressive DNA motifs to be a molecular ligand typical of commensals, supporting the notion that a balance between stimulatory and regulatory DNA motifs may contribute to the induction of controlled immune responses in the GI tract, thereby influencing the gut homeostasis maintenance [43]. The above-mentioned findings suggested that the endogenous regulatory DNA motifs abundant in specific commensal bacteria could serve as the core of DNA-based vaccines of therapeutic value.

### Effects of gut microbial metabolites on immunity

**Fig. 1:**
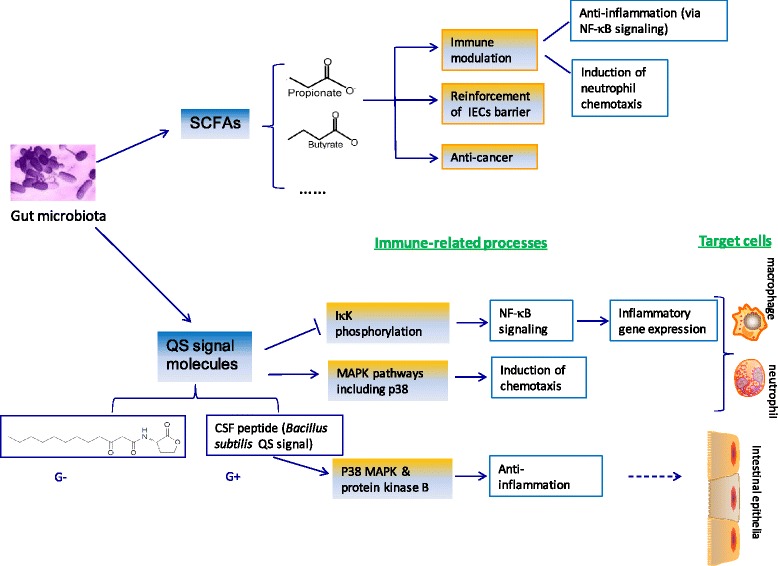
Gut microbial metabolites and host immune responses. CSF: Competence and sporulation factor; IECs: Intestinal epithelial cells. G^−^ and G^+^ indicate gram-negative and -positive bacteria, respectively

Gut microbiota-released metabolites, which are intermediates and/or end products of dietary constituents by commensal metabolism, may exert indispensable actions on host immunity and health [44]. Some of anaerobic gut microbes have the potential of converting dietary carbohydrates into organic acids including lactate, and short-chain fatty acids (SCFAs), the latter principally referring to acetate, propionate and butyrate. In mammals butyrate serves as a predominant energy substrate for colonocytes and enterocytes [45, 46]. Propionate is primarily absorbed by the liver while acetate is released into peripheral tissues [46]. In human gut, bacteria of the Bacteroidetes phylum secrete high levels of acetate and propionate whereas those of the Firmicutes phylum generate large amounts of butyrate [47]. Commensurate with increasing interests of SCFAs pertinent to Bacteroidetes and Clostridia phylum in the human gut [48], some other metabolites may serve as signaling molecules for inter-bacterial communication and quorum sensing. Among them are bacterial QS signals (also called autoinducers, or pheromones) and poly-γ-glutamic acid, the latter of which was recently characterized in *Bacillus subtilis*. Significant progress has been made to broaden our understanding about the modulatory effects of these gut microbial metabolites on host immunity (Fig. 1).

#### Short-chain fatty acids (SCFAs)

A growing body of evidence has revealed SCFAs as key metabolic and immune mediators [49, 50]. Distinct bioactivities of SCFAs may be attributed to their rapid absorption, with approximately only 5% being excreted through faeces. For instance, apart from the predominant energy source for the colonocytes, butyrate is found to be anti-inflammatory mainly through the suppression of NF-κB [51], be capable of altering the composition of the mucus layer by inducing mucin synthesis [52–54] and of exerting anti-cancer activities [55, 56]. Functional links are thus proposed among the dietary components, the gut microbiota composition and host immune homeostasis, inferring that different dietary preference may, at least partially, contribute to the racial and regional divergence in human population susceptibility to autoimmune disorders, inflammatory diseases and cancers.

Further studies with the experimental models of colitis and arthritis, have demonstrated that SCFAs could bind the GPR43 (G protein-coupled receptor 43, also known as free fatty acid receptor 2, FFAR2) and thus repressing the inflammation via interaction with FFAR2-expressing neutrophils [49, 57]. SCFAs, as endogenous ligands for the G-protein-coupled receptors GPR41 (viz. FFAR3) and GPR43 (viz. FFAR2), have been illustrated to mediate an array of metabolic processes such as the synthesis of glucagon-like peptide 1 in the enteroendocrine cells [45, 58].

There is ample evidence that SCFAs can activate GPR41 and GPR43 expressions in intestinal epithelial cells (ECs), leading to mitogen-activated protein kinase (MAPK) signaling, and production of chemokines and cytokines, which mediates protective immune response and tissue inflammation in mice [59]. The murine intestinal immune responses were investigated against immunological challenges including breach of the gut barrier (ethanol administration), 2, 4, 6-trinitrobenzene sulfonic-acid (TNBS) treatment, and infection of *Citrobacter rodentium*. GPR41 −/− and GPR43 −/− mice underwent the reduced inflammatory responses in the colon as indicated by low induction of inflammatory chemokines, cytokines and leukocyte infiltration. Furthermore, mice devoid of GPR41 or GPR43 failed to mount a normal Th1 response to TNBS treatment, which was in line with the notion derived from the ethanol administration that SCFA signals are indispensable for optimal acute inflammatory responses in the gut. The results clearly delineated beneficial roles of SCFAs and their receptors in conditioning gut ECs to mount prompt immunity in response to immunological stimuli in a GPR41- and GPR43-dependent manner [59].

Butyrate is widely recognized to be capable of inhibiting the expression of pro-inflammatory cytokines such as IL-12 and TNF-α [60, 61]. Butyrate is also demonstrated to induce the expression of intestinal epithelial heat shock protein (HSP) 25 and 72. Moreover, HSP 25 and 72, in addition to molecular chaperones, have been documented to be down-regulatory towards the expression of pro-inflammatory cytokines under stress such as infection and inflammation in the colon [62]. In contrast, either a fermentable fiber-lacking diet or chemical challenges mainly affecting anaerobic bacteria (by metronidazole administration), could manifestly decrease HSP expression in intestinal epithelia. In view of HSPs’ participation in the cellular responses to stressful factors and their hyper-expressions under inflammatory conditions, it has been postulated that butyrate may be associated with anti-inflammation.

Butyrate is known for its anti-inflammatory activities and thereby impacting host colon health [63, 64]. Accumulating evidence has shown that butyrate could attenuate bacterial translocation across epithelia under metabolic stress [65], and enhance the gut barrier via augmenting tight junction assembly [66]. In addition, a randomized, double-blind clinical trial has revealed the effects of butyrate as an adjunct therapy in combination with antibiotics on the treatment of shigellosis patients [67].

Propionate, derived from gut microbial fermentation of dietary inulin-type fructans (ITF, also known as a prebiotic nutrient), is reported to alleviate liver cancer cell proliferation [68]. As previously documented, ITF can alter the gut microbiota composition and activity [69]. In order to elucidate how ITF influenced neoplasm proliferation beyond the gut, researchers used mice transplanted with Bcr-Abl-transfected BaF3 cells receiving ITF supplementation. Ectopically Bcr-Abl-expressed pro-B murine BaF3 cells were chosen as the model under study because of their invasive and proliferative potentials in the lymphoid organs, such as liver tissues that could actively absorb the gut-originated SCFAs [70, 71]. The authors, by using gut microbiota analysis, in-vitro and in-vivo cell proliferation assays as well as serum SCFA quantitation, have in-vivo demonstrated that ITF attenuates hepatic BaF3 cell infiltration, increases propionate in the portal vein and lessens systemic inflammation. They have also in-vitro shown that propionate decreases BaF3 cell proliferation through a cAMP-dependent pathway and that activation of FFAR2 (viz. GPR43) alters proliferation of BaF3 and other human cancer cell lines. These data represent the first report that gut microbiotal conversion of prebiotic nutrients (ITF herein) into propionate could inhibit malignant cell proliferation beyond the gut.

Accumulating evidence indicates that a diverse range of commensal microbes could shape the gut immune system. It has been reported that colonization with *Clostridia* induces differentiation of peripheral Treg cells that have a critical role in the suppression of inflammatory and allergic responses [72, 73]. However, the molecular cues of such microbe-mediated Treg induction remain unknown. Two recent Nature papers demonstrate that the colonic microbial fermentation product butyrate tremendously enhances the differentiation of colonic Treg cells and thus meliorates colitis, which is dependent on an augmented histone H3 acetylation at the *Foxp3* promoter [74, 75]. As widely known, butyrate, and, to a lesser degree, propionate, are histone deacetylase (HDAC) inhibitors that epigenetically regulate gene expression. In the above-mentioned studies, propionate shows a moderate effect on extrathymic Treg cell induction. These findings suggest butyrate to be an inducer of extrathymic Treg cells in the colonic mucosa, and provide molecular insight into how a metabolite of gut microbiotal origin can modulate the cross-talk between commensal community and host immune system for gut homeostasis maintenance.

SCFAs including propionate and butyrate can activate gluconeogenesis (IGN) via complementary mechanisms. Intestinal IGN is known to mediate host glucose and energy homeostasis [45]. De Vadder et al. [45] illustrated that butyrate was able to activate IGN gene expression via a cAMP-dependent mechanism, whereas propionate, a substrate of IGN as well, could stimulate IGN gene expression via a gut-brain neural circuit involving the fatty acid receptor FFAR3. Conversely, in spite of similar modifications in gut microbiota composition, the SCFA-induced positive effects on body weight and glucose control observed with normal mice are abrogated in IGN-deficient mice. Altogether, regulation of IGN is essential for the metabolically beneficial roles of SCFAs and soluble fiber [45]. Despite the metabolic benefits being ascribed to fiber-rich diets in the past decades, this work unravels that IGN may contribute to favorable actions of SCFAs on body weight and glucose control [45].

#### Quorum sensing signals

Quorum sensing (QS), one of bacterial regulatory mechanisms to perceive and promote synchronized behaviors, relies on bacterial population density. This cell density-dependent system operates through the secreted small-molecular compounds called QS signals [76], which is utilized by pathogens to initiate the expression of virulence factors and biofilm formation and thereby facilitating their invasion and colonization into hosts [77, 78]. Evidence has revealed that such QS signals may also act as an important anti-immune arsenal and key mediators of inter-kingdom (host-bacteria) antagonistic relations [78, 79].

Host responses to pathogens involve the innate and adaptive immune reactions, both of which are committed to limit diffusion of the invaders. Notwithstanding, in order to control the probable detrimental consequences of pathogens, a variety of host regulatory elements may be operative including Tregs. Mucosal CD103+ DCs are known contributors to the conversion of Tregs depended on TGF-β and retinoic acid [80, 81].

*Pseudomonas aeruginosa*, an opportunistic pathogen, is a causative agent for diseases like cystic fibrosis, and often accounts for life-threatening nosocomial infections among immunocompromised individuals [82, 83]. *P. aeruginosa* produces more than one class of QS signals to coordinate its pathogenesis. In *P. aeruginosa* two chemically distinct classes of QS signals are identified to be N-acylhomoserine lactones (AHLs) and 4-hydroxy-2-alkylquinolines (HAQs) [84, 85]. Among them N-(3-oxododecanoyl)-L-homoserine lactone (3O-C_12_-HSL) is produced via the LasI synthase and sensed via the transcriptional activator LasR, which in turn modulates the expression of virulence factors and enhances biofilm maturation [86]. Ample evidence has revealed the involvement of *P. aeruginosa* 3O-C_12_-HSL in both establishment of bacterial pathogenesis and subversion of host immune system, suggestive of its immunosuppressive effects [86]. Kravchenko et al. [87] reported that the bacterial (*P. aeruginosa*) 3O-C_12_-HSL could selectively impair the regulation of NF-κB functions in activated mammalian cells, specifically dampening the induction of NF-κB–responsive genes that encode inflammatory cytokines and other immune modulators [87]. Their results demonstrate, for the first time, the anti-inflammatory effects of bacterial 3O-C_12_-HSL via in-vivo modulation of host NF-κB pathway, which likely contributes to the establishment and maintenance of local persistent infection of bacteria.

In addition to the well-studied AHLs, HAQs-the second class of *P. aeruginosa* QS signals encompass the derivatives of 4-hydroxy-2-heptylquinoline (HHQ) and the corresponding dihydroxylated derivatives such as 2-heptyl-3,4-dihydroxyquinoline (PQS, pseudomonas quinolone signal) [84]. Regulatory effects of HAQs were investigated in the host innate immunity using a wild-type (PA14) and two mutants of *P. aeruginosa*. Results have unraveled that bacterial HHQ and PQS could actively inhibit innate immune responses in vitro and in vivo via the NF-κB pathway. Specifically, HHQ and PQS were found to attenuate the NF-κB binding to its binding sites and to downregulate the expression of NF-κB target genes, and PQS was also observed to delay the degradation of IκB (inhibitor of κB) [84]. The above-mentioned work provides a paradigm that bacterial suppression of host immune system by QS signals is an effective strategy for bacterial immune evasion and survival in the hostile host environment.

Mounting evidence has shown the effects of bacterial AHLs on neutrophils, macrophages and other mammalian cells. Human neutrophils are found to be attracted by QS molecules 3O-C_12_-HSL and -C_10_-HSL to the sites of infection and developing biofilms [88]. It appears that human primary neutrophils can strongly be stimulated by 3O-C_12_-HSL and -C_10_-HSL in a dose-dependent manner, with no distinct effects being displayed in the case of C_4_-HSL supplementation [88]. Mechanisms were further explored whereby these QS signals were able to induce chemotaxis in human neutrophils. Results revealed that these long- and middle-chain fatty acid AHLs could act through Ca mobilization and actin remodeling, suggesting AHLs as key mediators during the recruitment of inflammatory cells to the infection sites [88].

Given the human phagocytic cell-activating and in-vitro polymorphonuclear neutrophils (PMN)-chemotactic potentials of 3O-C_12_-HSL, further studies have been conducted to investigate how 3O-C_12_-HSL activates neutrophils and to analyze signaling pathways relevant to migration [89]. The work focused on the mitogen activated protein (MAP) kinase p38 because an inhibitor of p38 (SB203580) was known to prevent the 3O-C_12_-HSL-mediated chemotaxis. Data showed that 3O-C_12_-HSL swiftly induced activation of the MAP kinase p38, which in turn activated MAPKAP-Kinase 2 (MK2) and its target, the leukocyte specific protein1 (LSP1), the latter being able to directly interact with F-actin. LSP1 was activated (phosphorylated) and co-localized with F-actin in polarized PMN upon exposure to 3O-C_12_-HSL, suggesting that: (1) 3O-C_12_-HSL might induce p38-dependent chemotaxis; (2) the p38 signaling is functionally linked to the cytoskeleton dynamics via LSP1 [89].

QS molecule 3O-C_12_-HSL plays critical roles in not only inter-bacterial communication but inter-kingdom signaling. It is believed that the ability of 3O-C_12_-HSL to downregulate the production of TNF-α (key proinflammatory cytokine) in stimulated macrophages may contribute to the establishment of chronic infections by such opportunistic bacteria as *P. aeruginosa* [90]. The authors (2013) showed that, in contrast to the suppression of TNF-α secretion, 3O-C_12_-HSL could amplify the production of major anti-inflammatory cytokine IL-10 in lipopolysaccharide (LPS)-stimulated murine RAW264.7 macrophages as well as peritoneal macrophages [90]. Furthermore, 3O-C_12_-HSL could increase IL-10 mRNA levels and IL-10 promoter reporter activity in LPS-stimulated RAW264.7 macrophages, indicating its modulatory effects on IL-10 at the transcriptional level. Finally, 3O-C_12_-HSL could remarkably potentiate the LPS-stimulated NF-κB DNA-binding levels and prolong p38 MAPK phosphorylation in RAW264.7 macrophages, suggesting that the increased transcriptional activity of NF-κB and/or p38-activated transcription factors might upregulate IL-10 production in macrophages upon exposure to both LPS and 3O-C_12_-HSL. These findings collectively unravel another circuit of the complex array of host transitions whereby opportunistic bacteria down-regulate host immune responses to thrive and to establish a chronic infection.

In addition to QS signals produced by G^−^ bacteria such as *P. aeruginosa*, those derived from Gram-positive (G^+^) bacteria are found to exert immunomodulatory actions on hosts [91]. A kind of QS signal from *Bacillus subtilis*, also termed competence and sporulation factor (CSF), has been demonstrated to be stimulant of the key survival pathways including p38 MAP kinase and protein kinase B (Akt) in mammalian intestinal epithelial cells [92]. Moreover, CSF seems to induce HSPs for protecting intestinal epithelial cells from oxidant stress and for avoiding the loss of barrier function. The intestinal homeostasis-maintenance ability of CSF is found to rely on its absorption by an apical membrane organic cation transporter-2 (OCTN2). Accordingly, the finding of OCTN2-mediated CSF transport unravels a new aspect of host–bacterial interactions that facilitates host monitoring and responding to behavioral or compositional changes of colonic microbiota. More recently, the same group investigated the *B. subtilis*-originated CSF by determining its impacts on attenuating intestinal inflammation. Results showed that anti-inflammatory effect of CSF was mediated by the downregulation of pro-inflammatory mediators (IL-4, IL-6 and CXCL-1), the upregulation of anti-inflammatory cytokine IL-10, and the induction of cytoprotective protein HSPs in Caco-2/bbe cells (human intestinal epithelial cell). The histological score of intestinal inflammation in 2% dextran sodium sulfate (DSS)-treated mice under the administration of 10nM CSF was distinctly lower than that in control mice. Additionally, CSF was observed to be able to ameliorate the survival ratio of mice formerly treated with a lethal dose of DSS. It is thus concluded that CSF may represent one of potential therapeutic strategies for intestinal inflammation [92].

Pathogen-secreted QS signals may influence the migration and activation of intestinal DCs. Bacterial 3O-C_12_-HSL and *Pseudomonas* quinolone signal (PQS) are validated to participate in tuning DC programs to regulate T cell effector function, which acts by lowering IL-12 production of DCs without altering their IL-10 release [93]. This suggests that 3O-C_12_- HSL and PQS would drive the maturation pattern of stimulated DCs awry from a pro-inflammatory T-helper type I (Th1) response and thereby decreasing the antibacterial activity of the adaptive immune defense. Thus 3O-C_12_-HSL and PQS seem to possess dual activities during the process of infection —— inducers of virulence factors, and immune-modulators facilitating the persistent infection of pathogen.

Certain infectious diseases have been demonstrated to hinder the onset of autoimmune disorders as observed with animal models, suggesting the probable impacts of these infectious agents in pathology of mammalian autoimmune diseases. Small molecules/proteins isolated from the infectious agents have shown to account for these protective effects [94]. Previous studies indicated that *P. aeruginosa* QS signal OdDHL (viz. 3O-C_12_- HSL) could delay the onset of type 1 diabetes (T1D) in the non-obese diabetic (NOD) mouse model. Furthermore, using an antigen-presenting cell-free system, the authors showed that 3O-C_12_-HSL could not only inhibit the proliferation of naïve T cells but directly suppress the differentiation of T cell subsets; however, no effects was seen with 3O-C_12_-HSL on the inhibition of primed and committed differentiated T cell responses, suggesting that 3O-C_12_-HSL-mediated immune mechanism may be restricted to initial stages of infection [94].

Gut-residing nonpathogenic *Escherichia coli* may secrete QS signals including autoinducer 2 (AI-2). In view of AI-2’s relevance as a bacterial signaling molecule, its actions in HCT-8 cells (intestinal epithelial cells, IEC) were recently investigated [95]. Inflammatory cytokine IL-8, a key player in attracting neutrophils, was found to be initially upregulated at all levels of AI-2 examined at 6 and 12 h post-treatment, followed by a distinct down-regulation at 24 h post-treatment. Collectively, nonpathogenic bacterial QS signal AI-2, is likely an IEC signaling molecule and may stimulate the transcription of immune-associated pathways, followed by the upregulation of negative-feedback elements that may block the inflammatory responses.

Gut microbes may produce metabolites other than SCFAs and QS signaling molecules, for instance, poly-γ-glutamic acid (γ-PGA) during fermentation of soybeans. Gamma-PGA is present predominantly in *Bacillus subtilis* but absent in mammals [96]. Studies have demonstrated that *Bacillus*-originated γ-PGA can regulate Th1/Th2 cell development depending on APC, specifically by stimulating DCs to favor the polarization of naïve CD4+ T cells toward Th1 rather than Th2 cells, and it also controls Th17 cell development through APC-dependent and -independent mechanisms [96].

There is evidence to show that *Bacillus*-derived γ-PGA may signal naïve CD4+ T cells to promote selective differentiation of Treg cells and to repress the differentiation of Th17 cells [97]. The initiation of FoxP3 expression by γ-PGA was partially attributed to TGF-β induction via a TLR-4/myeloid differentiating factor 88 (MyD88)-dependent pathway; however, this pathway was dispensable for γ-PGA suppression of Th17 differentiation. Intriguingly, in-vivo supplementation of γ-PGA was found to be able to attenuate symptoms of experimental autoimmune encephalomyelitis (EAE), concurrent with the declined Th17 cell infiltrations in the central nervous system. Therefore, γ-PGA was characterized as a type of the microbe-associated molecular pattern (MAMP), and also a novel mediator of autoimmune responses that enables the selective differentiation of anti-inflammatory Treg cells and dampens the differentiation of proinflammatory Th17 cells. The above finding is reminiscent of the previous demonstration in the murine model that exposure to γ-PGA could suffice to alleviate Th2-mediated allergic asthma, likely by activating DCs to favor the induction of Th1 over Th2 cells [98]. Altogether, these results may underpin the therapeutic potential of γ-PGA in the Th17-dominated autoimmune disorders [97].

### Commensals and gut homeostasis

#### Commensal-induced Tregs mediate immunopathology

Intestinal commensal microbiota have been shown to modulate conventional T cell and Treg responses that are required for effective host defense against pathogens while circumventing autoimmune responses and other immunopathologic consequences. The presence of Treg cells can normally prevent inappropriate T cell responses towards commensal bacteria that may otherwise lead to inflammatory diseases.

*Bifidobacterium infantis* 35624 strain, originally isolated from human gastrointestinal mucosa, has received much attention in the past decade. Supplementation of commensal *B. infantis* 35624 was reported to induce the generation and function of Treg cells that control excessive NF-κB activation in mice, thereby contributing to host homeostasis maintenance and conferring protection from improper activation of the innate immunity against a translocating and spreading pathogen like *Salmonella typhimurium* [99]. Further studies by the same group demonstrated that administration of this commensal to healthy human volunteers could result in the augmented numbers of Foxp3 T cells and enhanced secretion of peripheral blood mononuclear cell IL-10 [100]. It is known that microbiota-DC interactions are able to induce Treg cells. *B. infantis*-stimulated human DCs were observed to induce Foxp3 and IL-10 secreting T cells [100]. Generally speaking, DC subsets, referring to monocyte-derived DCs (MDDCs), myeloid DCs (mDCs) and plasmacytoid DCs (pDCs), use different pattern recognition receptors to coordinate the Treg cell induction, Specifically, MDDC IL-10 and mDC IL-10 secretions were relied on TLR-2 and retinoic acid, whereas IL-10 secretion by pDC was dependent on TLR-9 and required indoleamine 2, 3-dioxygenase (IDO) [100].

Commensal microbiota have been validated to contribute to the homeostatic proliferation of Foxp3^−^ conventional CD4^+^ T cells and Foxp3^+^ Tregs [101]. Under long-term antibiotic administration, a manifest decline of conventional CD4^+^ T cell proliferation was detected in a systemic pattern whereas Foxp3^+^ Treg proliferation was observed to be locally distributed in gut-draining mesenteric lymph nodes and Peyer’s patches. Moreover, the proliferative response to microbial components was not mediated by TLRs as various TLR- and MyD88-deficient mice exhibited normal or even elevated conventional T cell and Foxp3^+^ Treg proliferation. Taken together, commensal microbiota-derived stimuli are able to promote the cycling of both conventional CD4^+^ T and Foxp3^+^ Treg cells, irrespective of TLR signaling.

An elaborately-designed study illustrated that a complex mixture of 46 strains of *Clostridium*, in particular *Clostridium* clusters IV and XIVa, could induce TGF-β in intestinal epithelial cells to intensify the subsequent accumulation of IL-10-producing induced T regulatory (iTreg) cells, which were known to suppress colitis in a DSS-challenged colitis model [72]. Certain *Clostridium* species, rather than *Lactobacillus* or *Bacteroides* ones, were found to suffice to increase the frequency of Foxp3^+^ Treg cells in the colon when transferred into germ-free (GF) mice. Consequently, oral administration of *Clostridium* during the early life of conventionally-raised mice might confer resistance to colitis and systemic IgE responses in adult mice, pinpointing a novel approach to treating autoimmunity and allergy [72].

It is becoming evident that the diversity and composition of commensal microbiota in human intestines may influence the equilibrium of conventional T and Treg cells, thereby modulating host gut immunity.

#### Commensal bacteria and the barrier function of intestinal epithelium

The mammalian digestive tract has evolved and developed a variety of attributes to defense against microbial infection. A monolayer of columnar epithelial cells, termed intestinal epithelial cells (IECs), connects each other via tight junctions, and is known to line the small and large intestines as well as the Peyer’s patch regions. The tight junctions are thought to limit the diffusion of moieties between epithelial cells [102]. IECs, as a barrier between the intestinal lumen and host connective tissues, are continuously subjected to numerous immunologic stimuli [60]. Commensals are believed to promote the generation and maturation of organized gut-associated lymphoid tissues (GALTs) because they facilitate recruitment of immune cells to the mucosa [14]. Evidence has revealed that the GALTs and other lymphoid tissues are poorly developed in GF mice, however, this deficiency could be rectified by the inoculation of conventional flora or oral supplementation of TLR ligands, which indicates that: (1) signals/products derived from the commensals play indispensable roles in the development of immune tissues; (2) TLR signaling is essential for the maturation of the developing immune system [103].

An aberrant epithelial barrier may primarily be involved in chronic inflammatory disorders and even cancers [104]. Impaired epithelial integrity is demonstrated to activate the resident inflammatory cells in response to pathogenic invaders or endogenous ligands, which, coupled with a failure of normal regulatory mechanisms that limit leukocyte activation, would initiate a cascade leading to chronic inflammation [104]. In addition, the integrity of the epithelial barrier relies on homeostatic regulatory mechanisms involving mucosal induction of Treg cells, where commensal-host interactions undoubtedly play a role. Secretory IgA (SIgA) are believed to orchestrate with innate defense components for protecting the epithelium and strengthening its barrier function [105]. Segmented filamentous bacteria (SFB), a class of anaerobic and clostridia-related spore-forming commensals present in the gut of mammals (i.e. mice and humans), are found to be intimately attached to the epithelial lining of the mammalian GI tract [106, 107], and to actively interact with immune system [107]. SFB inoculation into GF mice has been validated to induce the production of SIgA and the recruitment of intraepithelial lymphocytes (IEL) to the gut [73, 108]. Work with immunocompetent mice has delineated that, intestinal SFB colonization is able to promote the production of mucosal SIgA, the differentiation of effector T helper 1 (Th1), effector T helper 2 (Th2) and Th17 cells, and the development of Treg cells [109]. Previous experimental data revealed that IEL, particularly γδIEL, might be involved in the regulation of the generation and differentiation of IECs [110]. Collectively, SFB is likely to closely participate in the regulation of IEC proliferation, suggesting its contribution to the barrier functionality of intestinal epithelium.

Another paradigm of gut commensal that affects gate-keeper functionality of epithelia is believed to be *Akkermansia muciniphila* [111]. *A. muciniphila* possessing mucin-degrading activity is a dominant human bacterium colonizing in the mucus layer of gut. The presence of *A. muciniphila* was demonstrated to be inversely correlated with body weight in mice and humans [111]. Administration of *A. muciniphila* appears to elevate the intestinal levels of endocannabinoids that controls inflammation, the gut barrier, and gut peptide secretion. A hypothesis has been proposed that *A. muciniphila* may play a crucial role in the mutualism between the gut microbiota and host, which regulates gut barrier function and other physiological functions during obesity and type 2 diabetes (T2D). Furthermore, merely viable *A. muciniphila* is able to exert the above-described actions because supplementation of heat-killed cells failed to improve the metabolic profile or to enhance the mucus layer thickness [111].

#### Commensal bacteria modulate gut homeostasis

Previous studies have revealed that *Bacteroides thetaiotaomicron*, a dominant member of gut microflora in mice and human, has potential of triggering the development of intestinal submucosal capillary network [112]. Angiogenesis stimulation by *B. thetaiotaomicron* was illustrated to be driven through bacteria-sensing Paneth cells in the epithelial crypt. Paneth cells, a key component of the intestinal innate immunity, are known to secrete an arsenal of antimicrobial peptides and proteins into the gut lumen [113]. Indigenous inhabitant *B. thetaiotaomicron* is thus pinpointed to be involved in both the mucosal barrier reinforcement and immune modulation.

The colonization of SFB, as previously described in the context of barrier functionality of intestinal epithelium, may also direct post-natal maturation of the gut mucosal lymphoid tissue, trigger a potent and broad IgA response, stimulate the T-cell compartment, and upregulate intestinal innate defense mediators, suggesting immune-stimulatory capacities of SFB [114, 115]. Apart from their abilities to educate the gut immune system, it becomes evident that SFB colonization may act as an adjuvant on systemic responses and thereby exacerbating pathologies in the murine models of encephalitis and arthritis, while conferring the genetically-predisposed mice protection from the development of T1D [98, 116–118]. SFB are thought to be species between obligate and facultative symbionts due to their high auxotrophic demands as evidenced by genomic sequencing of these symbionts with the rodents. These findings collectively suggest that SFB may benefit, at least nutritionally, from their interaction with the host and have thus evolved adaptive strategies to cope with host immune responses for maintaining their intestinal niches [119–121]. By using SFB-host cell co-cultivation system, Schnupf and co-workers [107] unraveled that single-celled SFB isolated from monocolonized mice underwent morphologic development and differentiation to release viable infectious particles, termed the intracellular off-springs, which enabled their colonization within the host for the induction of signature immune responses. In-vitro studies further demonstrated that those intracellular off-springs possessed the capabilities of attaching to host cells and of recruiting actin. Moreover, the up-regulations of host innate defense genes, inflammatory cytokines, and chemokines were found to be elicited by SFB [107].

New studies by Littman group [122] reported that, after inoculation of SFB, differentiation of Th17 cells was induced during which the IL-22 production by type 3 innate lymphoid cells (ILC3) was required for potentiating epithelial secretion of serum amyloid A (SAA). Moreover, while “poised-state” T cells expressing the Th17 main regulator RORγt (RORγt + Th17) were distributed throughout the gut, IL-17-expressing Th17 cells were limited to the small intestine ileum, coincided with the site of SFB adhering to epithelium. Another independent work by Atarashi et. al. illustrated that this preferential induction of IL-17 in Th17 cells might be attributed to intimate SFB attachment to the small intestine epithelium [123]. Overall, these recent findings have revealed a novel circuit of epithelial cell perception of intestinal commensals like SFB, the latter of which could modulate host immune responses including cytokine production, thereby facilitating our further exploitation of roles of Th17 cells in the regulation of mucosal defenses and control of autoimmune diseases.

Microbiota, by establishing inter-connected metabolic/nutritional networks and developing biofilms among their components, are able to confine the resources to potential pathogens that out-compete well-adapted indigenous microbes for ecological niches [124]. In addition to the occupation of ecological niches by commensals, documented are other mechanisms such as homeostasis-maintenance of commensals towards host. Studies have demonstrated the capabilities of non-virulent bacteria *Lactobacillus* spp., *Bacteroides* spp., and *Escherichia coli* to suppress poly-ubiquitylation and subsequently degrade IκB–α, which in turn inhibits the NF-κB activation and thereby leading to immune hypo-responsiveness in the intestines [125]. Supporting this finding, *B. thetaiotaomicron* was validated to stimulate the export of RelA (p65 subunit of NF-κB) from the host nucleus, which lowered the transcription of NF-κB-dependent genes [126]. Moreover, *Lactobacillus casei* was shown to exert anti-inflammatory actions through repressing the degradation of the inhibitor of NF-κB (IκB) as well [127]. Subsequent studies with *L. casei* DG (a probiotic strain) revealed that rectal administration of *L. casei* DG coupled with 5-aminosalicylic acid (5-ASA), rather than 5-ASA in combination with oral administration of this probiotic strain, could alter colonic microbiota composition by increasing *Lactobacillus* spp. and declining *Enterobacteriaceae*. In addition, this approach remarkably reduced the levels of TLR-4 and IL-1β mRNA while increasing mucosal IL-10. Accordingly, modification of mucosal microbiota by *L. casei* DG and its impacts on the mucosal immunity seem to be critical for the favorable roles of this probiotics in ulcerative colitis patients [128].

Another independent study presents the induction of host Treg cells and mucosal tolerance by *Bacteroides fragilis* capsular polysaccharide (PSA) [129]. The underlying mechanism may be related to the perception of *B. fragilis-*released PSA by host DCs through TLR2, which results in elevated production of Treg cells and anti-inflammatory cytokines and thereby contributing to colitis alleviation [129]. The finding of outer membrane vesicles (OMVs)-associated PSA not only reveals immunomodulatory effects of *B. fragilis* but also represents a novel mechanism regarding inter-kingdom cross-talk between the commensal and mammalian cells mediated by a bacterial molecule.

**Fig. 2:**
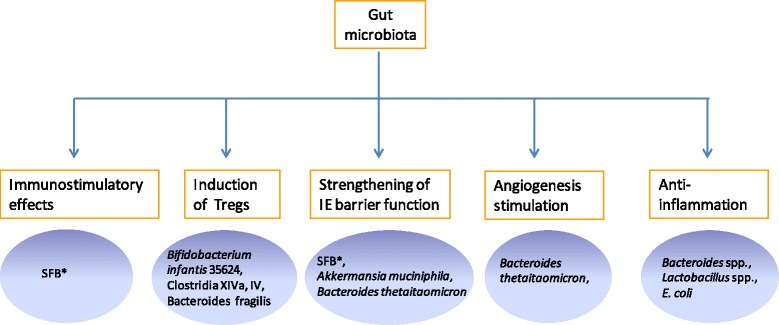
Commensals and gut homeostasis. *Segmented filamentous bacteria (SFB) also possess immunostimulatory effects, including induction of SIgA response, post-natal maturation of gut-associated lymphoid tissue (GALT), and stimulation of T cell compartment. IE: intestinal epithelium

It is believable that more and more immunomodualtory commensals will be unveiled owing to the advances in our research techniques such as gnotobiotic cultivation, comparative metagenomics/meta-proteomics approach, deep sequencing, microbiome studies, metabolomics related systems biology studies, *in-situ* 3D imaging, molecularly biological and immunologic methods, thereby deepening our understandings of the mechanisms underlying the interaction of commensal-host immune system. In addition to the documented effects of commensals on gut homeostasis (Fig. 2), the anticipated findings of commensals, most of which may fall into unculturable clades, would shed light on our novel therapeutic regimen to treat autoimmune disorders and inflammation associated with dysbiosis in human intestine.

#### Inter-species signals among commensals in the gut

An equilibrium among the gut, its beneficial microbiota (commensals) and pathogens is vital for human health, which represents an outcome of intricate and finely-tuned communication between microbes and host as well as that of cross-talk among microbes. Indole, present at high amounts (250–1100 μM) in the gut, probably serves as an inter-kingdom signal during the interactions of commensals and host intestinal cells [78].

Previous work demonstrated that indole, secreted by commensal *E. coli*, could lower the chemotaxis, motility, and adherence of pathogenic *E. coli* to host intestinal epithelial cells [130]. Furthermore, exposure to physiologically relevant levels of indole was found to up-regulate the genes associated with the mucosal barrier reinforcement and mucin production, which was in line with an elevation in the trans-epithelial resistance of the human enterocyte HCT-8 cells. In addition, indole was validated to decline the indicators of inflammation, such as the TNF-α-mediated NF-κB activation, the expression of proinflammatory IL-8, and to attenuate the attachment of pathogenic *E. coli* to HCT-8 cells; conversely, it could elevate the expression of anti-inflammatory IL-10. Analogous to the observations with probiotics strains, this study strongly suggested that commensal-secreted indole could serve as a beneficial signaling molecule for intestinal epithelial cells and thus be crucial in the protective responses to gut pathogens [130].

An independent investigation with a murine model has revealed the association between commensal-derived indole and enhanced epithelial barrier function. GF mice exhibited a reduced expression of junctional complex molecules in colonic ECs. Oral administration of indole-containing capsules was observed to cause an elevated expression of both tight junction (TJ)- and adherens junction (AJ)-associated molecules in colonic ECs of GF mice. In accordance with the increased expression of these junctional complex molecules, GF mice treated with indole were found to display an enhanced resistance against DSS-induced colitis. Protective potential of indole from DSS-induced epithelial insults was found in the GF mice as well as in the specific pathogen-free (SPF) mice. Altogether, the findings suggest the involvement of gut commensal-derived indole in the epithelial barrier enhancement in the colon [131].

There is evidence to reveal that glucagon-like peptide-1 (GLP-1) secretion from murine enteroendocrine cells is modified by the exposure of indole at similar level to that detected in the human large intestine [132]. Strikingly, indole was observed to elevate the release of GLP-1 during short exposure time but mitigate GLP-1 secretion over longer time. The dual effects of indole were thought to involve two key molecular mechanisms in intestinal enteroendocrine L cells. Indole, on the one hand, could suppress voltage-gated K^+^ channel, elevate the temporal width of action potentials provoked by L cells, and result in the increased Ca^2+^ entry, thereby triggering abrupt GLP-1 secretion. On the other hand, indole could reduce ATP production by blockage of NADH dehydrogenase and thus leading to a lasting decline of GLP-1 secretion. Accordingly gut microbiota-originated indole is regarded to have a remarkable effect on host metabolism, underpinning indole as a signaling molecule that mediates the communication of gut microbiota with enteroendocrine L cells [132].

Indole is widely recognized to regulate versatile aspects of indole-producing bacteria, such as spore formation [133], plasmid stability [134], drug resistance [135], biofilm formation [136, 137], and virulence [138]. Interestingly, besides indole-producers, indole also influences several physiological traits in non-indole-producing bacteria. For instance, *Salmonella enterica* serovar Typhimurium, a gut pathogen unable to produce indole, relies on indole in its drug resistance and virulence as evidenced by the down-regulations of host cell invasion-related genes, and of bacterial flagellum production upon indole exposure [139]. Indole, present in the gut commensal consortium, has been validated to be a key signaling molecule for inter-species communication to control drug resistance and virulence of *S. enterica*, a causal agent for human gastroenteritis, bacteremia, and typhoid fever [140].

A delicately-designed study was conducted regarding population dynamics during the development of antibiotic-resistant *E. coli* strains [141]. A continuous culture of *E. coli* was performed under the exposure to increased levels of antibiotic. Less resistance was observed for a large majority of the above isolates than the overall *E. coli* population. There was evidence to reveal that few highly-resistant mutants could enhance the survival of the less-resistant *E. coli* cells within the same population, partially by indole, which is a bacterial signal produced by unstressed and robustly-growing *E. coli* cells. Indole was known to transcriptionally activate drug efflux pumps and to trigger protective mechanisms under oxidative stress. Within the population, synthesis of indole might come at a fitness cost to the highly-resistant bacterial isolates, which is achieved by drug-resistance mutations irrelevant to indole synthesis as determined by whole-genome sequencing. Accordingly this work underpins that a population-based resistance mechanism may constitute a form of kin selection by which a minority of resistant mutants can, at certain cost, endow protection to other more susceptible cells and thereby promoting the survival of the entire population under unfavorable conditions including antibiotics stress [141].

Besides indole itself, its derivative indole-3-acetonitrile (IAN) has also been shown to affect the virulence of opportunistic pathogen *C. albicans* by attenuating the fungal attachment to HT-29 intestinal epithelial cells, and by inhibiting fungal filamentation and biofilm formation [142]. Moreover, indole and IAN could specifically stimulate the transcription of NRG1, the transcriptional repressor that influences *C. albicans* pathogenesis. The work further adopted the model host *Caenorhabditis elegans* to in-vivo illustrate that the exposure to indole or IAN could suppress fungal infection and reduce *C. albicans* colonization in the nematode gut. This was in line with a previous demonstration that extracellular indole was able to activate genes in association with *Vibrio* polysaccharide (VPS) production, as well as to influence the expression of various bacterial genes relative to virulence, transport, iron utilization and motility, indicative of indole as a signal in *Vibrio* [143].

More recently, indole 3-propionic acid (IPA), another derivative of indole, is reported to cause the down-regulation of TNF-α in enterocytes and the up-regulation of junctional protein-coding mRNAs while acting as an in-vivo ligand for pregnane X receptor (PXR), the xenobiotic sensor [144]. PXR has previously been characterized to be a mediator in microbial indole-dependent regulation of host intestinal barrier function [144]. In their work, manifestly leaky intestinal epithelia were observed concurrent with the up-regulated TLR signaling pathway in PXR deficient (Nr1i2 −/−) mice. Furthermore, the above-mentioned epithelial barrier leakage was abolished in Nr1i2−/− Tlr4−/− mice. Therefore a direct chemical communication has been proposed between the intestinal symbionts and PXR to regulate mucosal integrity through an indole signaling pathway in intestines [144]. Indole is widely accepted as a key player in ecological balance, bacterial physiology, and possibly human health [145]. Overall, evidence to date suggests a rational that indole and indole-related signaling molecules may be indispensable in the inter-kingdom regulatory networks pertinent to intestinal health.

### Microbiota and metabolic disorders

**Fig. 3:**
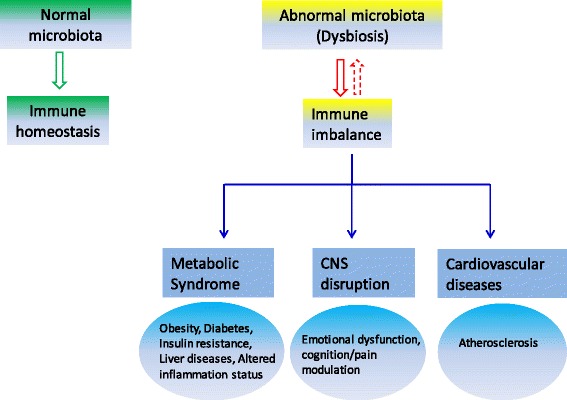
Effects of gut microbiota on the peripheral tissues beyond the gut. CNS: central nervous system

From the metabolic viewpoint, gut microbiota may be recognized as a consortium capable of modulating host physiology and immunity [146]. Gut microbes impact local and systemic inflammation through pattern recognition receptors (PRRs) [147, 148]. Accumulating evidence has revealed that gut microbes may regulate fat mass expansion via their fermentative products and mediate the suppression of the fasting induced adipose factor [69, 149–152]. Intestinal dysbiosis, referring to “alterations in the composition and abundance of the gut microbiota as compared to healthy individuals” [153], is believed to account for inflammatory, metabolic diseases and even dysfunctions of central nervous system (Fig. 3).

#### Diabetes and obesity

It is thought that commensals are able to exert crucially biological actions on their host tissues, ranging from metabolic regulations to immune-modulations. Any unequilibrium between the host and commensals would lead to the passage of the luminal contents into the underlying tissues and thus into the bloodstream, triggering the immune response activation and the ensuing gut inflammation, which may contribute to various diseases including infectious enterocolitis, IBD, obesity, diabetes, irritable bowel syndrome, small intestinal bacterial overgrowth, hepatic fibrosis, food intolerances and atopic manifestations [154].

##### T1D and autoimmunity

Data heretofore underpin the evolving theory that gut microbiota serve as an organ with a myriad of previously neglected or poorly-understood metabolic, immunologic, and endocrine-like effects on human health [155]. An evident correlation has been validated between the altered intestinal microbiota composition with the onset of autoimmune disorders such as T1D [156]. Gut microbiota is found to participate in the progression of early incidence of T1D, which is originated from T-cell-mediated destruction of insulin-producing pancreatic *β*-cells. Experimental data suggest that dialogue between gut microbiota and host innate immunity is closely associated with islet destruction [156, 157]. Consequently, the gut microbiota-innate immunity axis is proposed to be crucial in the development of T1D.

Accumulating evidence from human and animal models suggests environmental cues (including the human microbial milieu) may be indispensable in T1D etiology [158]. The substantially rising incidence of T1D in recent decades is found in very young children worldwide, particularly in the developed countries. Children who progressed to T1D had decreased richness of Firmicutes and increased Bacteroidetes over time whereas the situation is the opposite for age-matched healthy children (with increased Firmicutes and decreased Bacteroidetes). In contrast to children with ongoing autoimmunity, healthy children harbored a more diverse and stable intestinal microbiome [157]. Studies with non-obese diabetic (NOD) mice have shown that their incidence of spontaneous T1D could be affected by the microbial milieu in the animal housing facility or by exposure to microbial stimuli such as administration with mycobacteria or various microbial products [158, 159].

The infant gut exhibits a Th2-skewed cytokine profiling that favors triggering immunological ignorance toward bacterial and dietary components [160]. Hansen et al. [160] tested the impacts of vancomycin (an antibiotic that inhibits biosynthesis of G^+^ bacterial cell wall) on the early microbial colonization of the gut by administrating the drug at neonatal stage of mice. Results showed that vancomycin depleted many major genera of G^+^ and G^−^ bacteria whereas one species, *Akkermansia muciniphila*, was not affected rather became dominant. Furthermore, overall diabetes incidence was found to be evidently lower in the neonatally vancomycin-treated mice than untreated controls, whereas the blood glucose levels significantly lower in the mice treated as adults than the other groups. In addition, an increase in cluster of differentiation CD4^+^ T cells producing pro-inflammatory cytokines was observed in the neonatally vancomycin-treated mice. Taken together, it is suggested that the early postnatal period would be critical for microbial protection from T1D, and *A. muciniphila* is considered to be a beneficial bacterium to protect the host from T1D onset, particularly at infancy [160].

MyD88 protein, an adaptor for multiple innate immune receptors that recognize microbial stimuli, is widely accepted to be one of the major signaling molecules participating in the activation of TLR (except TLR3) [161]. Studies have indicated that no T1D onset is observed in specific SPF NOD mice devoid of MyD88 protein [158]. The manifestation could be attributed to commensal microbiota because: (1) GF MyD88-deficient NOD mice developed distinct diabetes; (2) T1D was mitigated after colonization of these GF MyD88-deficient NOD mice with a defined bacterial phylum of healthy gut. The authors also illustrated that depletion of MyD88 could lead to alteration in the composition of the distal gut microbiota, and that exposure to the microbiota of SPF MyD88-deficient NOD donors might alleviate T1D in GF NOD recipients. Consequently, interaction of the intestinal microbiota with the innate immunity may be a key player in the epigenetic modulation of T1D susceptibility [158].

There is a long-time plausible theory termed hygiene hypothesis, meaning that a decline of early childhood exposure to microbes (both pathogenic and symbiotic) increases the susceptibility of autoimmune disorders by suppressing natural development of immune system, resulting in defective Treg cell induction and the ensuing loss of self-tolerance. This hypothesis has evolved and led to the rational that gut microbiotal alteration could be one of predisposing factors for the onset and development of autoimmunity such as T1D.

Recent work by Toivonen et al. [162] revealed the association of fermentable fibers (FF) with risk of T1D development using NOD mice. Their results showed that NOD mice fed with FF-free semisynthetic diets were distinctly protected from diabetes, whereas the FF-rich semisynthetic diet-fed counterparts displayed increased incidence of T1D. This manifestation was found to be correlated to the alterations in gut microbiota composition as evidenced by more dominating Bacteroidetes and reduced Firmicutes at phylum level in NOD mice supplied by FF-rich meal than those by FF-free meal. The high diabetogenic potential of FF, in particular of pectin and xylan, was linked to colonic expression of proinflammatory and stress-associated genes [162]. This taxonomic shift in gut microbiota associated with high risk of T1D incidence, Bacteroidetes dominating at phylum level compared to Firmicutes, is reminiscent of the documented features in individuals with Crohn’s disease [163], which is one of autoimmune disorder in human GI tract. Another study proposed that the characteristic manifestation of T1D —high Bacteroidetes to Firmicutes ratio, a lack of butyrate-producing bacteria, reduced bacterial diversity and weak community stability— occurred after the appearance of autoantibodies, suggesting the possible involvement of intestinal microbiota in the progression from pancreatic β-cell autoimmunity to clinical disorder but not in the onset of disease process [164].

Although the exact mechanism about local tolerance induction by the microbiota remains elusive, the finding that the normal intestinal microbiota could attenuate the progression of autoimmune T1D in a MyD88-independent manner would provide a different viewpoint into disease etiology. Rational utilization of live microbial strains or microbial products thereof may represent new therapeutic promises for T1D [157]. Continued endeavor to define the specific role of intestinal microbiome (the collective genomes of microbiota) in the onset of T1D is urgently needed for the design and development of novel disease preventative or therapeutic regimen.

##### T2D and obesity

Apart from T1D, extensive studies show that the intestine microbes affect host energy harvest in mammals, suggesting a link of gut microbiota with obesity [155]. *Firmicutes*, *Bacteroidetes*, *Actinobacteria* and *Proteobacteria* are known to dominate the human intestinal microbiota of adults [155]. It is well accepted that host body habitus is relevant to the composition of the gut microbiota. Ley et al have analyzed the microbiome of lean (ob/+ or +/+) mice in comparison to that of their obese (ob/ob) siblings which are homozygous for a mutation in the leptin gene with the resultant phenotype of severe obesity [158]. Analogous to human, *Firmicutes* and *Bacteroidetes* are predominant bacterial phyla in healthy murine intestines. The ob/ob mice are characterized by an increased prevalence of *Firmicutes* and a reduced abundance of *Bacteroidetes* as compared to lean sibling mice. Additionally, the microbiome of obese mice appears to be more efficient in energy harvest, as evidenced by the lower amount of energy remaining in the feces of obese mice than that in lean controls [152].

A pioneering work demonstrates the association of type 2 diabetes (T2D) with the translocation of commensal bacteria [161]. The authors presented that, during the early onset of high-fat diet(HFD)-induced diabetes, live commensal intestinal bacteria were actively translocated through intestinal mucosa into blood and the mesenteric adipose tissue (MAT) where they triggered a low-degree bacteremia [161]. The translocation relied on the microbial PRRs CD14 and Nod1 because no translocation was observed in mice devoid of CD14 or Nod1; however, it was elevated in Myd88-deficient and ob/ob mice. This metabolic bacteremia was definitive of an augmented co-localization with DCs from the intestinal lamina propria and of an elevated intestinal mucosal adherence of non-pathogenic *E. coli*. In addition, this manifestation could be rectified by 6-week probiotics administration with *Bifidobacterium animalis* subsp. lactis 420, a strain known to promote the mammalian inflammatory and metabolic status. This work proposed, for the first time, a new therapeutic regimen for the metabolic disease — intestinal bacterial adherence, bacterial translocation, or receptors of bacterial fragments could be promising targets to preventing/or inverting the incidence of diabetes and obesity [161]. This finding also broadens the avenue for treatment of metabolic disorders using probiotics strategies.

#### Liver diseases

Extensive studies with animal models have indicated that: (1) progression of chronic liver diseases like liver fibrosis relies on gut bacteria-derived products [165, 166], which may be attributed to the increased intestinal permeability; (2) bacterial translocation is a crucial player in fibrosis progression during the development of chronic liver disorders [167]. However, the precise mechanisms underlying the mucosal barrier breach or bacterial translocation remain elusive. The microbiota is believed to be required for liver homeostasis in chronic liver injury [167]. A low baseline level of bacterial products is postulated to enable hepatic protection from toxic factors as supported by: (1) the down-regulation of hepatic expression of P450 enzymes (i.e. Cyp26a1) in the GF mice after chronic liver injury; (2) more vulnerability of hepatocytes to toxin-induced cell death in Myd88/Trif-deficient mice devoid of downstream TLR signaling than wild-type controls. Notably, higher systemic levels of microbial products are unable to provide additional resistance, but activate hepatic stellate cells and Kupffer cells/recruited macrophages to exacerbate liver damage. Furthermore, microbial-derived indole-3-propionic acid (IPA) might confer hepatic protection from oxidative stress. Altogether, the commensal microbiota endows prevention against fibrosis upon chronic liver injury in mice, representing a novel potential therapeutic regimen for liver diseases.

An elaborately-designed study has delineated the link between inflammasome-mediated dysbiosis and progression of non-alcoholic fatty liver disease (NAFLD) [168]. NAFLD is a prevalent chronic liver disorder in the developed countries, 20% of which may be proceeded to chronic hepatic inflammation (non-alcoholic steatohepatitis, NASH), the latter being associated with cirrhosis, portal hypertension and hepatocellular carcinoma. However the precise mechanism underlying the progression from NAFLD to NASH remains to be elucidated. Henao-Mejia and colleagues have demonstrated that the NLRP6 and NLRP3 inflammasomes and their effector IL-18 can negatively regulate NAFLD/NASH progression as well as multiple aspects of metabolic syndrome by modulating the gut microbiota [168]. Alterations in the gut microbiota configuration in mice are observed under deficiency of inflammasomes, which links to exacerbated hepatic steatosis and inflammation via influx of TLR4 and TLR9 agonists into the portal circulation, resulting in the enhanced hepatic TNF-α expression that promotes NASH progression. Moreover, exacerbation of hepatic steatosis and obesity were observed while inflammasome-lacking mice were co-housed with wild-type controls. Taken together, changes in the gut microbiota-host interaction derived from the impairment in NLRP3 and NLRP6 inflammasome sensing, may govern the propensity for development of multiple metabolic syndrome-associated abnormalities such as NAFLD-NASH progression [168]. The above findings would shed light on the crucial role of the microbiota in the pathogenesis of systemic auto-inflammatory and metabolic disorders that are seemingly irrelevant. Analogous to metabolic disorders (i.e. obesity), an increased prevalence of the Phylum *Firmicutes* is definitive of gut dysbiosis following the onset of toxic liver diseases including liver fibrosis and NASH [168, 169], which underpins the involvement of gut microbiota in the metabolic and immunologic aspects of human health.

#### Inflammation tones

The functional links between gut microbiota and inflammation/metabolic diseases have recently been illustrated [170, 171]. Peroxisome proliferator-activated receptor γ (PPARγ) is a well-recognized transcription factor to link metabolism to inflammation in the intestine*.* In addition to the GI tract, PPARγ is predominantly expressed in adipose tissue and thus participating in the metabolic regulation of lipids, glucose homeostasis, cell proliferation and differentiation as well as local inflammation. In this regard, it is not surprising that microbiota-induced PPARγ is able to exert various actions beyond the gut, as evidenced by its regulatory effects on the expression of Angiopoietin like protein-4 (Angptl 4), the latter of which is responsible for lipid storage in the adipose tissues [149, 172, 173]. *Streptococcus salivarius*, one of the primo-colonizers of human oral and gut mucosal surfaces, had previously been shown to influence inflammation by down-regulating NF-κB in the intestinal cells [174], and has recently been revealed to inhibit transcriptional activity of PPARγ [172]. Upon exposure to *S. salivarius* supernatant, the expression levels of I-FABP (intestinal fatty acid binding protein) and Angptl 4 were found to markedly drop among PPARγ-induced metabolic genes in the IECs [172]. Altogether, data strongly suggest that *S. salivarius* could exert dual effects on both host inflammatory regulation and metabolism processes. This is reminiscent of the past demonstration regarding PPARγ-dependent anti-inflammatory mechanism induced by nonpathogenic *B. thetaiotaomicron* that selectively antagonizes NF-κB. It appeared that *B. thetaiotaomicron* might target NF-κB subunit RelA by enhancing its nuclear export through a mechanism independent of nuclear export receptor Crm-1. PPARγ, together with nuclear RelA, underwent nucleocytoplasmic redistribution upon exposure to *B. thetaiotaomicron* [126]. A decline in PPARγ is able to abrogate both the nuclear export of RelA and anti-inflammatory effect of *B. thetaiotaomicron* [126].

Of particular interest, an ameliorating role of the gut microbiota has been demonstrated with BaF3 mice, a murine leukemia model established by transplantation of BaF3 cells containing ectopic expression of Bcr-Abl oncogene and characteristic of cachectic symptoms such as loss of fat mass, muscle atrophy, anorexia and inflammation at the final stage [70]. The gut microbial 16S rDNA analysis unraveled a dysbiosis and selective modulation of *Lactobacillus* spp. (decline of *L. reuteri* and *L. johnsonii/gasseri* in favor of *L. murinus*/*animalis*) in the BaF3 mice as compared to controls. Restoration of *Lactobacillus* spp. by oral administration with *L. reuteri* 100-23 and *L. gasseri* 311476 was found to decrease the expression of atrophy markers (Atrogin-1, MuRF1, LC3, Cathepsin L) in the gastrocnemius and in the tibialis, a manifestation correlated with a drop of inflammatory cytokines (IL-6, monocyte chemoattractant protein-1, IL-4, granulocyte colony-stimulating factor). These beneficial effects are thought to be strain- and/or species-specific since no impacts on muscle atrophy markers and systemic inflammation were seen with *L. acidophilus* NCFM administration under study [70]. The aforementioned work collectively suggests the gut microbiota as a promising therapeutic target in the control of leukemia-associated inflammation and relevant disorders in the muscle.

#### Central nervous system (CNS) and gut microbiome

A growing body of evidence has indicated that alterations of the gut microbiome may result in dysregulation of immune responses in both the gut and the distal effector immune sites including the central nervous system (CNS). Recent studies in experimental autoimmune encephalomyelitis (EAE) — an animal model of human multiple sclerosis, have revealed that modifying certain intestinal bacterial populations may cause a pro-inflammatory manifestation that in turn contributes to the onset and development of autoimmune diseases, for example, human multiple sclerosis. Conversely, some commensal bacteria and their antigenic products, while presented in the correct context, can protect against inflammation within the CNS [175].

The mechanism governing this bi-directional communication between CNS and gut microbiome is postulated to be related to microbial endocrinology—the ability of bacteria to respond to as well as to produce the same neurochemicals acting as neurohormones in the host [176]. The field of microbial endocrinology was established several decades ago when the term was initially coined by Lyte [177, 178].

The ability of microbiota to produce neurochemicals with hormonal activities suggests that influences of microbiome interacting with host may not merely be confined to the intestines, rather go beyond the gut. Studies with GF animals and animals receiving pathogenic challenges, probiotic strains or antibiotic medications, have unveiled important roles of gut microbiota in the modulation of anxiety, depression, cognition and pain [176, 179–182].

In human subjects with irritable bowel syndrome (IBS), which are characteristic of altered microbial composition and diversity as compared to healthy controls, emotional dysfunctions were found including anxiety and depression [183]. An augmentation in the nerve-to-mast cell proximity in the colonic mucosa of IBS patients has been demonstrated to be correlated with the severity and frequency of abdominal pain [179].

Cryan and colleagues [180] have shown that *Lactobacillus rhamnosus*, a probiotic lactic acid bacterium, could pose direct effects on murine emotional behaviors like anxiety and depression, probably by mediating neurotransmitter (GABA) receptors [180]. Moreover, the neurophysiological effects of *L. rhamnosus* were abolished in the vagotomized mice, indicating the vagal signaling as a predominant constitutive communication pathway for modulating the bacteria-brain interplay. Dietary alterations such as feeding of meat, which could substantially vibrate the composition of the microbiome, have been illustrated to promote memory and learning in mice [181].

In addition to bacterial biosynthesis and sensing of similar neurohormones found in the mammals, it is postulated that the bacterial symbiosis with the intestine plays a predominant role in the postnatal development and maturation of the host immune and endocrine system, which underpins CNS function [182]. Recent studies have demonstrated that host microbiota controls maturation and function of microglia in the CNS [184]. Microglia, as the tissue macrophages of the brain, are crucial for maintaining tissue homeostasis, for scavenging dying cells/components, and for eradicating pathogens through microbial associated molecular pattern receptor-dependent and -independent mechanisms [185]. Apart from their aforementioned functions, microglia are central to axon pruning and remodeling during development and adulthood, indicating them as an essential player in brain development [186].

Host microbiota are manifestly associated with microglia homeostasis, as supported by the GF mice displaying global defects in microglia with altered cell percentages and an immature phenotype which leads to impaired innate immune responses. Limited microbiota complexity or temporal depletion of microbiota might contribute to defective microglia in mice. Reintroduction of a complex microbiota into the murine host could, to some degree, rectify microglia properties [184]. Furthermore, SCFAs, a category of gut microbiotal products as described earlier in this review, were found to be able to modulate microglia homeostasis [184]. Thus it is not surprising that mice lacking the SCFA receptor FFAR2 would mimic the impaired microglia as previously observed in GF mice [184].

### Impaired immune system impacts intestinal microbiota composition

The mucosal immune system –— constituting adaptive, innate immune cells and the epithelium –— is considerably affected by its microbial milieu [187]. There is accumulating evidence to reveal *vice versa*, that the impaired immune system influences the intestinal microbiota composition and in turn links to diseases.

Disruption of innate immune pathways is able to cause alterations in intestinal microbiota. For instance, Nod2, a kind of PRRs in response to bacterial muramyl dipeptide, is associated with susceptibility to Crohn’s disease. Analysis of intestinal bacteria from the terminal ilea of Nod2-lacking mice demonstrated an increased load of commensal resident bacteria. Furthermore, Nod2-deficient mice abolished the capability of preventing pathogenic bacterial colonization from intestine [188]. Another independent study showed that Nod2 could profoundly influence the early development and composition of the intestinal microbiota [189]. Apart from Nods, TLRs — an essential part of innate immunity — may influence intestinal microbiota as well. TLR5, expressed in the gut mucosa, is recognized to participate in the defense against pathogens. Recent experimentation with TLR5-lacking mice has revealed dysbiosis (with over-numbered *E. coli*) and the ensuing chronic inflammation, which in turn leads to manifestation of metabolic syndrome [187, 190].

Investigation with MyD88−/− NOD mice also supports the aforementioned rational that impaired immune system affects the intestinal microbiota composition. MyD88−/− NOD mice possessed high abundance of Bacteroidetes, and thereby circumventing the host from T1D development [158]. Furthermore, more SFB were observed in the small intestines of MyD88−/− mice than wild-type counterparts [191], suggesting that the depletion of MyD88 signals could alter the intestinal microbiota composition.

Recent studies have shown that Crohn’s disease (CD) patients with impaired immune system displayed a declined biodiversity and prevalence of intestinal bacteria. Particularly, an elevated risk of post-surgery CD recurrence is linked up with a decreased abundance of *Faecalibacterium prausnitzii* on resected ileal Crohn mucosa [192]. *F. prausnitzii*, a prominent member of Firmicutes and absent in CD patients’ microbiota, was demonstrated to have anti-inflammatory effects in vitro and in vivo. In Caco-2 cells, *F. prausnitzii*’s released metabolites other than butyrate, could hamper the activation of NF-κB and secretion of IL-8. Furthermore, in peripheral blood mononuclear cells (PBMCs), *F. prausnitzii* was able to induce a declined release of proinflammatory cytokines IL-12 and IFN-γ, and an increased secretion of anti-inflammatory cytokine IL-10. The spectrum of cytokines secreted by PBMCs has been documented as an indicator to assess the in-vitro and in-vivo immunomodulatory potential of various bacterial strains [193]. Therefore *F. prausnitzii* could drive the immune responses toward Th2 pathway. *F. prausnitzii* displayed a greater IL10/IL12 ratio than *L. salivarius* Ls33, a known anti-inflammatory probiotic strain, indicating *F. prausnitzii* as a more potent probiotic species. Its in-vitro effects were confirmed in vivo on TNBS-induced colitis murine model. The aforementioned data collectively showed that the dysbiosis associated with TNBS-induced colitis (concurrent with impaired immune system) was partially rectified by *F. prausnitzii* or its supernatant [192].

Acquired immune deficits could affect the microbiota composition as exemplified by patients with human immunodeficiency virus (HIV) infection. Transmission, propagation and persistence of HIV are thought to occur largely in the host mucosal tissues [194]. Rapid depletion of mucosal memory CD4^+^ T cells is observed during acute infection and is sustained throughout the chronic phase of disease, concurrent with a decline in the Th17/Tregs ratio, an increase in CD8^+^ T cells, and a drop in the NK cells. Moreover, HIV-infected subjects have elevated epithelial permeability, systemic microbial translocation, and increased serum inflammatory status, which are considered to propel the disease progression to AIDS [195, 196]. Recent studies have assessed the mucosal and fecal microbiome of treated HIV patients as compared to untreated ones, which substantiates the altered microbial flora in HIV patients [196–198]. The work by McCune’s group has investigated colonic microbiota composition of untreated viremic HIV-infected subjects in comparison to that of uninfected healthy controls [196]. It revealed that in HIV-infected individuals, *Erysipelotrichaceae* family was the most abundant, and *Enterobacteriaceae* the second most abundant including species of *Salmonella*, *Escherichia*, *Serratia*, *Shigella* and *Klebsiella* genera that are recognized proinflammatory pathobionts. In contrast, distinctly reduced abundance of genera of *Bacteroides* and *Alistipes* was found in HIV-infected subjects compared to healthy controls [196]. Another study with rectal mucosal microbiota reported the compositional shifts in association with HIV infection [198]. Relative abundance of *Fusobacteria*, *Anaerococcus*, *Peptostreptococcus* and *Porphyromonas* was significantly enriched in HIV-infected subjects not receiving combination anti-retroviral therapy (cART), while that of genera including *Roseburia*, *Coprococcus*, *Ruminococcus*, *Eubacterium*, *Alistipes* and *Lachnospira* was depleted [198]. HIV-positive subjects on cART displayed similar tendency of microbiota alterations (enrichment and depletion for every genus as above-described) to those of HIV-infected subjects not receiving cART, which were within the intermediate magnitude of variation and were not statistically significant relative to healthy controls [198].

Acquired immunodeficiency can also be observed in post-surgery patients receiving solid-organ transplantation and administrated with immunosuppressive agents. Transplant recipients were reported to have altered microbial composition in their salivary bacterial communities as evidenced by a rising proportion of pathobionts such as *Klebsiella*, *Acenitobacter*, *Staphylococcus*, *Enterococcus* and *Pseudomonas* [199]. Results revealed that immunosuppression did not significantly affect the dominating bacterial taxa (i.e. *Streptococcus, Prevotella*) but elevated the prevalence or relative abundance of taxa documented as opportunistic pathogens in immunocompromised hosts [199]. Among all the immunosuppressant drugs examined, only prednisone and mycophenolate mofetil showed a dose-dependent association with microbial colonization of oral cavity. In particular, prednisone dose was found to positively correlate with the prevalence of two genera *Klebsiella* and *Acenitobacter* [199].

## Conclusions

Intestinal microbiota is normally indispensable for shaping host gut immune system and thus contributing to gut homeostasis maintenance, and is also a key mediator in keeping metabolic functions in the peripheral tissues including liver and pancreas. Accumulating evidence has indicated that intestinal microbiota not only induces and reinforces pro-inflammatory immune responses but also elicits immunosuppressive responses. Abnormal microbial-elicited immunosuppression may result in dysregulation in host metabolism and/or impairment in anti-cancer immunity.

Data with regard to commensal bacteria have integrated, which leads up to a conclusion that a number of microbes are fluctuating on the boundary of virulence. *B. fragilis* is a representative of this phenomenon. This bacterium is able to improve the development of the host adaptive immune system while being confined to the lumen of the intestinal tract, but becomes enterotoxigenic while it contingently traverses the gut epithelial mucosa. Mazmanian et al [103] showed that during colonization of *B. fragilis* in animals, a bacterial polysaccharide A (PSA) was presented by DCs, which could direct and promote the maturation of the developing immune system [103]. Subsequent work by the same group substantiated the above finding and further explored the mechanisms of its immunomodulatory effects [129]. Not belonging to dominant members of the gut microbiota, *B. fragilis* is normally absent in conventionally raised SPF mice. Inoculation with *B. fragilis* has been found to protect mice from colitis in the T-cell-transferred and TNBS-treated animal models. It appeared that the purified *B. fragilis* PSA was sufficient to act on host analogous to the live bacterium, including the initiation of IL-10 production by Tregs, suppression of Th17 cell production, disease protection from colitis, and colonization of the host [129, 200]. On the other hand, *B. fragilis* is capable of producing Bft (*Bacteriodes fragilis* toxin), which acts indirectly by eliciting high levels of ROS and the ensuing damage of host DNA [201]. Sustained high-leveled ROS, once exceeding the host’s DNA repair capacity, may lead to DNA damage and thereby culminating in cell death or oncogenic mutations [202]. Thus *B. fragilis* is considered to be a risky factor for colorectal cancer in mammals. Such example also illustrates that a subtle balance is maintained between mammal hosts and microbial kingdom [203].

Mucosal surface barriers are essential for host-microbial symbiosis, the former of which are vulnerable to persistent microbial insults and dietary antigenic components, and must be repaired to re-establish homeostasis. Compromised flexibility of the host or microbiota may place itself on a “death tunnel” to malignancy [202]. In addition, manifestations that immunotherapies are displaying efficacy in malignancies of organs such as melanoma, bladder, renal and lung cancers rather than cancer of the colon, the latter being highly-populated by microbes, have garnered extensive attention as to whether and how the microbiota influences immunotherapy’s efficacy [202]. So the interplays of microbiota and immunotherapy efficacy/toxicity need further investigation.

Among the metabolic disorders, NAFLD, which is characteristic of hepatic triglyceride (TG) accumulation rather than being arisen from alcohol abuse, is linked up with ectopic fat accumulation, especially in the liver. T2D is characterized by persistent hyperglycemia. Pathophysiologic mechanisms of NAFLD and T2D in common are believed to be relevant to insulin resistance, lipotoxicity, and inflammation [171]. Insulin resistance is a multi-organ manifestation as observed at the level of the liver, muscle and adipose tissues. Moreover, adipose tissues and the liver can secrete proinflammatory cytokines. In addition to insulin resistance and inflammation, other risk factors may contribute to the elevated incidence of metabolic diseases including lifestyle (high-fat/sugar diets and poor physical activity), gut microbiota alterations and environmental pollutants.

Based on data heretofore, it is hypothesized that the gut microbiota may mediate the influence of lifestyle factors triggering development of NAFLD and T2D [171]. A metagenome-wide association study on 345 Chinese patients with T2D *versus* healthy individuals has revealed that T2D sufferers exhibited a moderate degree of gut microbial dysbiosis, referring to a dearth of some butyrate-producing bacteria and an elevation in some opportunistic pathogens [204]. As afore-described in Section of “Liver diseases”, an increased prevalence of *Firmicutes*, a representation of dysbiosis, is found to be linked to NAFLD [168, 169, 205]. Of particular interest, these two metabolic disorders, NAFLD and T2D, to some extent, share similar mechanisms of etiology: being associated with dysbiosis. These novel findings would broaden our knowledge about metabolic influences of a shifted intestinal microbiota beyond the gut and thus benefit our exploration of therapeutic targets for metabolic diseases.

Close to the completion of this manuscript, an interesting paper has been published demonstrating the link of atherosclerosis etiology with abnormal gut microbiota [206]. Studies with low-density lipoprotein receptor (LDLR) −/− mice, an atherosclerotic murine model, revealed that 12-week supplementation of high-fat diet could lead to evident aortic lesions, macrophage infiltration, and collagen level increase, concurrent with an up-regulation of inflammatory factors [206]. This finding suggests that gut microbiota, combined with metabolisms of fatty acids and vitamin B_3_, could play a profound role in the onset and development of atherosclerosis [206] (Fig. 3).

A growing body of novel “omics” technologies based on next-generation sequencing, nuclear magnetic resonance (NMR) spectroscopy and gas chromatography coupled with flame ionization detector/mass spectrometry (GC–FID/MS) is gaining wide popularity in the field of cardiometabolic diseases in association with microbiota dysbiosis. The integration and comparison of omics-mode data and molecular biological data would offer comprehensive insight into the mechanisms by which microbiota and metabolites thereof influence host immunity and metabolism. Commensal microbiota in the intestine may serve as a consortium with immunologic and endocrine-like activities to modulate the epigenetic status of host cells. Owing to the advances in genome-wide epigenetic analysis, for instance, chromatin immunoprecipitation sequencing (CHIP-Seq), researchers can determine and analyze these epigenetic modifications, thereby deciphering the intrinsic intestinal microbiota–host interactions and unraveling the impacts of microbiota within and beyond the gut such as liver, cardiovascular system, and even CNS.

## Abbreviations

(gamma-)γ-PGA: Poly-γ-glutamic acid
3O-C_12_-HSL: N-(3-oxododecanoyl)-L-homoserine lactone
AHLs: N-acylhomoserine lactones
Bft: *Bacteriodes fragilis* toxin
CD: Crohn’s disease
CHIP-Seq: Chromatin immunoprecipitation sequencing
CNS: Central nervous system
CpG: Cytidine-phosphate-guanosine
DAMP: Damage-associated molecular pattern
DCs: Dendritic cells
DSS: Dextran sodium sulfate
EAE: Experimental autoimmune encephalomyelitis
ECs: Epithelial cells
FFAR2: Free fatty acid receptor 2
GALTs: Gut-associated lymphoid tissues
GC−FID/MS: Gas chromatography coupled with flame ionization detector/mass spectrometry
GF: Germ-free
gfDNA: gut-floral-derived DNA
GI: Gastrointestinal
GLP-1: Glucagon-like peptide-1
GPR 41/43: G protein-coupled receptor 41/43
HAQs: 4-hydroxy-2-alkylquinolines
HFD: High-fat diet
HHQ: 4-hydroxy-2-heptylquinoline
HSP: Heat shock protein
IAN: Indole-3-acetonitrile
IBS: Irritable bowel syndrome
IDO: Indoleamine 2, 3-dioxygenase
IECs: Intestinal epithelial cells
IPA: Indole 3-propionic acid
ITF: Inulin-type fructans
iTreg: induced T regulatory cells
LDLR: Low-density lipoprotein receptor
LPS: Lipopolysaccharide
LSP1: Leukocyte specific protein 1
MAPK: Mitogen activated protein kinase
MAT: Mesenteric adipose tissue
mDCs: myeloid DCs
MDDCs: Monocyte-derived DCs
MyD88: Myeloid differentiating factor 88
NAFLD: Non-alcoholic fatty liver disease
NASH: Non-alcoholic steatohepatitis
NMR: Nuclear magnetic resonance
NOD: Non-obese diabetic
OCTN2: Organic cation transporter-2
ODN: Oligonucleotiodes
PAMPs: Pathogen-associated molecular patterns
PBMCs: Peripheral blood mononuclear cells
pDCs: plasmacytoid dendritic cells
PPARγ: Peroxisome proliferator-activated receptor γ
PQS: Pseudomonas quinolone signal
PRRs: Pattern recognition receptors
PSA: Polysaccharide A
PXR: Pregnane X receptor
QS: Quorum sensing
SCFAs: Short-chain fatty acids
SFB: Segmented filamentous bacteria
SIgA: Secretory IgA
SLE: Systemic lupus erythematosus
SPF: Specific pathogen-free
T1D: Type 1 diabetes
T2D: Type 2 diabetes
Teff: effector T
Th1: T-helper type I
TLRs: Toll-like receptors
TNBS: 2, 4, 6-trinitrobenzene sulfonic acid
Tregs: Regulatory T cells

## References

[1] Ley RE, Peterson DA, Gordon JI (2006). Ecological and evolutionary forces shaping microbial diversity in the human intestine. Cell.

[2] Round JL, Mazmanian SK (2009). The gut microbiota shapes intestinal immune responses during health and disease. Nat Rev Immunol.

[3] Atarashi K, Honda K (2011). Microbiota in autoimmunity and tolerance. Curr Opin Immunol.

[4] Chow J, Lee SM, Shen Y, Khosravi A, Mazmanian SK (2010). Host–Bacterial Symbiosis in Health and Disease. Adv Immunol.

[5] Loftus EV (2004). Clinical epidemiology of inflammatory bowel disease: Incidence, prevalence, and environmental influences. Gastroenterology.

[6] O’Neill LAJ (2008). The interleukin-1 receptor/Toll-like receptor superfamily: 10 years of progress. Immunol Rev.

[7] Paust S, Cantor H (2005). Regulatory T cells and autoimmune disease. Immunol Rev.

[8] Maldonado RA, von Andrian UH (2010). How tolerogenic dendritic cells induce regulatory T cells. Adv Immunol.

[9] Bermudez-Brito M, Munoz-Quezada S, Gomez-Llorente C, Matencio E, Bernal MJ, Romero F (2013). Cell-free culture supernatant of *Bifidobacterium breve* CNCM I-4035 decreases pro-inflammatory cytokines in human dendritic cells challenged with *Salmonella typhi* through TLR activation. PLoS One.

[10] Steinman RM (2012). Decisions about dendritic cells: Past, present and future. Annu Rev Immunol.

[11] Cools N, Petrizzo A, Smits E, Buonaguro FM, Tornesello ML, Berneman Z (2011). Dendritic cells in the pathogenesis and treatment of human diseases: a Janus Bifrons?. Immunotherapy.

[12] Shen C, He Y, Cheng K, Zhang D, Miao S, Zhang A, Meng F, Miao F, Zhang J (2011). Killer artificial antigen-presenting cells deplete alloantigen-specific T cells in a murine model of alloskin transplantation. Immunol Lett.

[13] Banchereau J, Briere F, Caux C, Davoust J (2000). Immunobiology of dendritic cells. Annu Rev Immunol.

[14] Macpherson AJ, Harris NL (2004). Interactions between commensal intestinal bacteria and the immune system. Nat Rev Immunol.

[15] Chang PV, Hao L, Offermanns S, Medzhitov R (2014). The microbial metabolite butyrate regulates intestinal macrophage function via histone deacetylase inhibition. Proc Natl Acad Sci U S A.

[16] Koh A, De Vadder F, Kovatcheva-Datchary P, Bäckhed F (2016). From Dietary Fiber to Host Physiology: Short-Chain Fatty Acids as Key Bacterial Metabolites. Cell.

[17] Kawasaki T, Kawai T, Akira S (2011). Recognition of nucleic acids by pattern-recognition receptors and its relevance in autoimmunity. Immunol Rev.

[18] Krieg AM, Vollmer J (2007). Toll-like receptors 7, 8, and 9: linking innate immunity to autoimmunity. Immunol Rev.

[19] Hall JA, Bouladoux N, Sun CM, Wohlfert EA, Blank RB, Zhu Q (2008). Commensal DNA limits regulatory T cell conversion and is a natural adjuvant of intestinal immune responses. Immunity.

[20] Fűri I, Sipos F, Germann TM, Kalmár A, Tulassay Z, Molnár B (2013). Epithelial toll-like receptor 9 signaling in colorectal inflammation and cancer: Clinico-pathogenic aspects. World J Gastroenterol.

[21] Krieg AM (2006). Therapeutic potential of Toll-like receptor 9 activation. Nat Rev Drug Discov.

[22] Lamphier MS, Sirois CM, Verma A, Golenbock DT, Latz E (2006). TLR9 and the recognition of self and non-self nucleic acids. Ann N Y Acad Sci.

[23] Blasius AL, Beutler B (2010). Intracellular Toll-like receptors. Immunity.

[24] Ries M, Schuster P, Thomann S, Donhauser N, Vollmer J, Schmidt B (2013). Identification of novel oligonucleotides from mitochondrial DNA that spontaneously induce plasmacytoid dendritic cell activation. J Leukoc Biol.

[25] Viglianti GA, Lau CM, Hanley TM, Miko BA, Shlomchik MJ, Marshak-Rothstein A (2003). Activation of autoreactive B cells by CpG dsDNA. Immunity.

[26] Crispin JC, Liossis SN, Kis-Toth K, Lieberman LA, Kyttaris VC, Juang YT (2010). Pathogenesis of human systemic lupus erythematosus: recent advances. Trends Mol Med.

[27] Ronnblom L, Pascual V (2008). The innate immune system in SLE: type I interferons and dendritic cells. Lupus.

[28] Barrat FJ, Meeker T, Gregorio J, Chan JH, Uematsu S, Akira S (2005). Nucleic acids of mammalian origin can act as endogenous ligands for Toll-like receptors and may promote systemic lupus erythematosus. J Exp Med.

[29] Zanetti M (2004). Cathelicidins, multifunctional peptides of the innate immunity. J Leukoc Biol.

[30] Lande R, Gregorio J, Facchinetti V, Chatterjee B, Wang YH, Homey B (2007). Plasmacytoid dendritic cells sense self-DNA coupled with antimicrobial peptide. Nature.

[31] Matzinger P (2002). The danger model: a renewed sense of self. Science.

[32] Rubartelli A, Lotze MT (2007). Inside, outside, upside down: damage-associated molecular-pattern molecules (DAMPs) and redox. Trends Immunol.

[33] Zimmer C (2009). Origins. On the origin of eukaryotes. Science.

[34] Mathew A, Lindsley TA, Sheridan A, Bhoiwala DL, Hushmendy SF, Yager EJ (2012). Degraded mitochondrial DNA is a newly identified subtype of the damage associated molecular pattern (DAMP) family and possible trigger of neurodegeneration. J Alzheimers Dis.

[35] Zhang Q, Raoof M, Chen Y, Sumi Y, Sursal T, Junger W (2010). Circulating mitochondrial DAMPs cause inflammatory responses to injury. Nature.

[36] Brinkmann V, Reichard U, Goosmann C, Fauler B, Uhlemann Y, Weiss DS (2004). Neutrophil extracellular traps kill bacteria. Science.

[37] Kwa S, Kannanganat S, Nigam P, Siddiqui M, Shetty RD, Armstrong W (2011). Plasmacytoid dendritic cells are recruited to the colorectum and contribute to immune activation during pathogenic SIV infection in rhesus macaques. Blood.

[38] Reeves RK, Evans TI, Gillis J, Wong FE, Kang G, Li Q (2012). SIV infection induces accumulation of plasmacytoid dendritic cells in the gut mucosa. J Infect Dis.

[39] Wang X, Xue L, Yang Y, Xu L, Zhang G (2013). TLR9 promoter polymorphism is associated with both an increased susceptibility to gastric carcinoma and poor prognosis. PLoS One.

[40] Kauppila JH, Karttunen TJ, Saarnio J, Nyberg P, Salo T, Graves DE (2013). Short DNA sequences and bacterial DNA induce esophageal, gastric and colorectal cancer cell invasion. APMIS.

[41] Fu B, Zhang Y, Long W, Zhang A, Zhang Y, An Y, Miao F, Nie F, Li M, He Y, Zhang J, Teng G (2014). Identification and characterization of a novel phage display-derived peptide with affinity for human brain metastatic breast cancer. Biotechnol Lett.

[42] Landrigan A, Wong MT, Utz PJ (2011). CpG and non-CpG oligodeoxynucleotides directly costimulate mouse and human CD4+ T cells through a TLR9- and MyD88-independent mechanism. J Immunol.

[43] Bouladoux N, Hall JA, Grainger JR, dos Santos LM, Kann MG, Nagarajan V (2012). Regulatory role of suppressive motifs from commensal DNA. Mucosal Immunol.

[44] Romani L, Zelante T, Palmieri M, Napolioni V, Picciolini M, Velardi A (2015). The cross-talk between opportunistic fungi and the mammalian host via microbiota’s metabolism. Semin Immunopathol.

[45] De Vadder F, Kovatcheva-Datchary P, Goncalves D, Vinera J, Zitoun C, Duchampt A (2014). Microbiota-generated metabolites promote metabolic benefits via gut-brain neural circuits. Cell.

[46] Guarner F, Malagelada JR (2003). Gut flora in health and disease. Lancet.

[47] Macfarlane S, Macfarlane GT (2003). Regulation of short-chain fatty acid production. Proc Nutr Soc.

[48] Flint HJ, Bayer EA, Rincon MT, Lamed R, White BA (2008). Polysaccharide utilization by gut bacteria: potential for new insights from genomic analysis. Nat Rev Microbiol.

[49] Maslowski KM, Vieira AT, Ng A, Kranich J, Sierro F, Yu D (2009). Regulation of inflammatory responses by gut microbiota and chemoattractant receptor GPR43. Nature.

[50] Neish AS (2009). Microbes in gastrointestinal health and disease. Gastroenterology.

[51] Segain JP, de la Raingeard Blétière D, Bourreille A, Leray V, Gervois N, Rosales C (2000). Butyrate inhibits inflammatory responses through NF-kappaB inhibition: implications for Crohn’s disease. Gut.

[52] Brown CT, Davis-Richardson AG, Giongo A, Gano KA, Crabb DB, Mukherjee N (2011). Gut microbiome metagenomics analysis suggests a functional model for the development of autoimmunity for type 1 diabetes. PLoS One.

[53] Burger-van Paassen N, Vincent A, Puiman PJ, van der Sluis M, Bouma J, Boehm G (2009). The regulation of intestinal mucin MUC2 expression by short-chain fatty acids: implications for epithelial protection. Biochem J.

[54] Finnie IA, Dwarakanath AD, Taylor BA, Rhodes JM (1995). Colonic mucin synthesis is increased by sodium-butyrate. Gut.

[55] Flint HJ, Scott KP, Louis P, Duncan SH (2012). The role of the gut microbiota in nutrition and health. Nat Rev Gastroenterol Hepatol.

[56] Scheppach W, Bartram HP, Richter F (1995). Role of short-chain fatty acids in the prevention of colorectal cancer. Eur J Cancer.

[57] Brown AJ, Goldsworthy SM, Barnes AA, Eilert MM, Tcheang L, Daniels D (2003). The orphan G protein-coupled receptors GPR41 and GPR43 are activated by propionate and other short chain carboxylic acids. J Biol Chem.

[58] Tolhurst G, Heffron H, Lam YS, Parker HE, Habib AM, Diakogiannaki E (2012). Short-chain fatty acids stimulate glucagon-like peptide-1 secretion via the G-protein coupled receptor FFAR2. Diabetes.

[59] Kim MH, Kang SG, Park JH, Yanagisawa M, Kim CH (2013). Short-chain fatty acids activate GPR41 and GPR43 on intestinal epithelial cells to promote inflammatory responses in mice. Gastroenterology.

[60] Artis D (2008). Epithelial-cell recognition of commensal bacteria and maintenance of immune homeostasis in the gut. Nat Rev Immunol.

[61] Magalhaes JG, Tattoli I, Girardin SE (2007). The intestinal epithelial barrier: how to distinguish between the microbial flora and pathogens. Semin Immunol.

[62] Arvans DL, Vavricka SR, Ren H, Musch MW, Kang L, Rocha FG (2005). Luminal bacterial flora determines physiological expression of intestinal epithelial cytoprotective heat shock proteins 25 and 72. Am J Physiol Gastrointest Liver Physiol.

[63] Louis P, Flint HJ (2009). Diversity, metabolism and microbial ecology of butyrate-producing bacteria from the human large intestine. FEMS Microbiol Lett.

[64] Pryde SE, Duncan SH, Hold GL, Stewart CS, Flint HJ (2002). The microbiology of butyrate formation in the human colon. FEMS Microbiol Lett.

[65] Lewis K, Lutgendorff F, Phan V, Soderholm JD, Sherman PM, McKay DM (2010). Enhanced translocation of bacteria across metabolically stressed epithelia is reduced by butyrate. Inflamm Bowel Dis.

[66] Peng LY, Li Z, Green RS, Holzman IR, Lin J (2009). Butyrate enhances the intestinal barrier by facilitating tight junction assembly via activation of AMP-activated protein kinase in Caco-2 cell monolayers. J Nutr.

[67] Raqib R, Sarker P, Mily A, Alam NH, Arifuzzaman AS, Rekha RS (2012). Efficacy of sodium butyrate adjunct therapy in shigellosis: a randomized, double-blind, placebo -controlled clinical trial. BMC Infect Dis.

[68] Bindels LB, Porporato P, Dewulf EM, Verrax J, Neyrinck AM, Martin JC (2012). Gut microbiota-derived propionate reduces cancer cell proliferation in the liver. Br J Cancer.

[69] Everard A, Lazarevic V, Derrien M, Girard M, Muccioli GM, Neyrinck AM (2011). Responses of gut microbiota and glucose and lipid metabolism to prebiotics in genetic obese and diet-induced leptin resistant mice. Diabetes.

[70] Bindels LB, Beck R, Schakman O, Martin JC, De Backer FC, Sohet FM (2012). Restoring specific lactobacilli levels decreases inflammation and muscle atrophy markers in an acute leukemia mouse model. PLoS One.

[71] Fiskus W, Pranpat M, Bali P, Balasis M, Kumaraswamy S, Boyapalle S (2006). Combined effects of novel tyrosine kinase inhibitor AMN107 and histone deacetylase inhibitor LBH589 against Bcr-Abl-expressing human leukemia cells. Blood.

[72] Atarashi K, Tanoue T, Shima T, Imaoka A, Kuwahara T, Momose Y (2011). Induction of colonic regulatory T cells by indigenous Clostridium species. Science.

[73] Umesaki Y, Setoyama H, Matsumoto S, Imaoka A, Itoh K (1999). Differential roles of segmented filamentous bacteria and clostridia in development of the intestinal immune system. Infect Immun.

[74] Arpaia N, Campbell C, Fan X, Dikiy S, van der Veeken J, deRoos P (2013). Metabolites produced by commensal bacteria promote peripheral regulatory T-cell generation. Nature.

[75] Furusawa Y, Obata Y, Fukuda S, Endo TA, Nakato G, Takahashi D (2013). Commensal microbe-derived butyrate induces the differentiation of colonic regulatory T cells. Nature.

[76] Keller L, Surette MG (2006). Communication in bacteria: an ecological and evolutionary perspective. Nat Rev Microbiol.

[77] Lin L, Tan RX (2011). Cross-kingdom actions of phytohormones: A functional scaffold exploration. Chem Rev.

[78] Rumbaugh KP, Kaufmann GF (2012). Exploitation of host signaling pathways by microbial quorum sensing signals. Curr Opin Microbiol.

[79] Bandyopadhaya A, Kesarwani M, Que Y-A, He J, Padfield K, Tompkins R (2012). The quorum sensing volatile molecule 2-amino acetophenon modulates host immune responses in a manner that promotes life with unwanted guests. PLoS Pathog.

[80] Coombes JL, Siddiqui KR, Arancibia-Cárcamo CV, Hall J, Sun CM, Belkaid Y (2007). A functionally specialized population of mucosal CD103+ DCs induces Foxp3+ regulatory T cells via a TGF-beta and retinoic acid-dependent mechanism. J Exp Med.

[81] Sun CM, Hall JA, Blank RB, Bouladoux N, Oukka M, Mora JR (2007). Small intestine lamina propria dendritic cells promote de novo generation of Foxp3 Treg cells via retinoic acid. J Exp Med.

[82] Chastre J, Fagon JY (2002). Ventilator-associated pneumonia. Am J Respir Crit Care Med.

[83] Rello J, Diaz E (2003). Pneumonia in the intensive care unit. Crit Care Med.

[84] Kim K, Kim YU, Koh BH, Hwang SS, Kim S-H, Lépine F (2010). HHQ and PQS, two *Pseudomonas aeruginosa* quorum-sensing molecules, down-regulate the innate immune responses through the nuclear factor-κB pathway. Immunology.

[85] Williams P, Winzer K, Chan WC, Camara M (2007). Look who’s talking: communication and quorum sensing in the bacterial world. Philos Trans R Soc B.

[86] Jadhav GP, Chhabra SR, Telford G, Hooi DSW, Righetti K, Williams P, Kellam B, Pritchard DI, Fischer PM (2011). Immunosuppressive but non-LasR-inducing analogues of the *Pseudomonas aeruginosa* quorum-sensing molecule N-(3-Oxododecanoyl)-L-homoserine lactone. J Med Chem.

[87] Kravchenko VV, Kaufmann GF, Mathison JC, Scott DA, Katz AZ, Grauer DC (2008). Modulation of gene expression via disruption of NF-κB signaling by a bacterial small molecule. Science.

[88] Karlsson T, Musse F, Magnusson K (2012). Vikström EN-Acylhomoserine lactones are potent neutrophil chemoattractants that act via calcium mobilization and actin remodeling. J Leukoc Biol.

[89] Kahle NA, Brenner-Weiss G, Overhage J, Obst U, Hänsch GM (2013). Bacterial quorum sensing molecule induces chemotaxis of human neutrophils via induction of p38 and leukocyte specific protein 1 (LSP1). Immunobiology.

[90] Glucksam-Galnoy Y, Sananes R, Silberstein N, Krief P, Kravchenko VV, Meijler MM (2013). The bacterial quorum-sensing signal molecule N-3-oxo-dodecanoyl-L-homoserine lactone reciprocally modulates pro- and anti-inflammatory cytokines in activated macrophages. J Immunol.

[91] Fujiya M, Musch MW, Nakagawa Y, Hu S, Alverdy J, Kohgo Y (2007). The *Bacillus subtilis* quorum-sensing molecule CSF contributes to intestinal homeostasis via OCTN2, a host cell membrane transporter. Cell Host Microbe.

[92] Okamoto K, Fujiya M, Nata T, Ueno N, Inaba Y, Ishikawa C (2012). Competence and sporulation factor derived from *Bacillus subtilis* improves epithelial cell injury in intestinal inflammation via immunomodulation and cytoprotection. Int J Color Dis.

[93] Skindersoe ME, Zeuthen LH, Brix S, Fink LN, Lazenby J, Whittall C (2009). *Pseudomonas aeruginosa* quorum-sensing signal molecules interfere with dendritic cell-induced T-cell proliferation. FEMS Immunol Med Microbiol.

[94] Gaisford W, Pritchard DI, Cooke A (2011). OdDHL inhibits T cell subset differentiation and delays diabetes onset in NOD mice. Clin Vaccine Immunol.

[95] Zargar A, Quan DN, Carter KK, Guo M, Sintim HO, Payne GF (2015). Bacterial secretions of nonpathogenic *Escherichia coli* elicit inflammatory pathways: a closer investigation of interkingdom signaling. MBio.

[96] Kim S, Yang JY, Lee K, Oh KH, Gi M, Kim JM (2009). *Bacillus subtilis-*specific poly-gamma glutamic acid regulates development pathways of naive CD4+ T cells through antigen-presenting cell-dependent and -independent mechanisms. Int Immunol.

[97] Lee K, Hwang S, Paik DJ, Kim WK, Kim JM, Youn J (2012). *Bacillus*-derived poly-γ-glutamic acid reciprocally regulates the differentiation of T helper 17 and regulatory T cells and attenuates experimental autoimmune encephalomyelitis. Clin Exp Immunol.

[98] Lee K, Kim S-H, Yoon HJ, Paik DJ, Kim JM, Youn J (2011). *Bacillus*-derived poly-γ-glutamic acid attenuates allergic airway inflammation through a Toll-like receptor-4-dependent pathway in a murine model of asthma. Clin Exp Allergy.

[99] O’Mahony C, Scully P, O’Mahony D, Murphy S, O’Brien F, Lyons A (2008). Commensal-induced regulatory T cells mediate protection against pathogen-stimulated NF-κB activation. PLoS Pathog.

[100] Konieczna P, Groeger D, Ziegler M, Frei R, Ferstl R, Shanahan F (2012). *Bifidobacterium infantis* 35624 administration induces Foxp3 T regulatory cells in human peripheral blood: potential role for myeloid and plasmacytoid dendritic cells. Gut.

[101] Cording S, Fleissner D, Heimesaat MM, Bereswill S, Loddenkemper C, Uematsu S (2013). Commensal microbiota drive proliferation of conventional and Foxp3+ regulatory CD4+ T cells in mesenteric lymph nodes and peyer’s patches. Eur J Microbiol Immunol.

[102] Kelly D, Mulder IE (2012). Microbiome and immunological interactions. Nutr Rev.

[103] Mazmanian SK, Liu CH, Tzianabos AO, Kasper DL (2005). An immunomodulatory molecule of symbiotic bacteria directs maturation of the host immune system. Cell.

[104] Karin M, Lawrence T, Nizet V (2006). Innate immunity gone awry: linking microbial infections to chronic inflammation and cancer. Cell.

[105] Brandtzaeg P (2013). Gate-keeper function of the intestinal epithelium. Benefic Microbes.

[106] Jepson MA, Clark MA, Simmons NL, Hirst BH (1993). Actin accumulation at sites of attachment of indigenous apathogenic segmented filamentous bacteria to mouse ileal epithelial cells. Infect Immun.

[107] Schnupf P, Gaboriau-Routhiau V, Gros M, Friedman R, Moya-Nilges M, Nigro G (2015). Growth and host interaction of mouse segmented filamentous bacteria in vitro. Nature.

[108] Talham GL, Jiang HQ, Bos NA, Cebra JJ (1999). Segmented filamentous bacteria are potent stimuli of a physiologically normal state of the murine gut mucosal immune system. Infect Immun.

[109] Gaboriau-Routhiau V, Rakotobe S, Lécuyer E, Mulder I, Lan A, Bridonneau C (2009). The key role of segmented filamentous bacteria in the coordinated maturation of gut helper T cell responses. Immunity.

[110] Komano H, Fujiura Y, Kawaguchi M, Matsumoto S, Hashimoto Y, Obana S (1995). Homeostatic regulation of intestinal epithelia by intraepithelial γδT cells. Proc Natl Acad Sci U S A.

[111] Everard A, Belzer C, Geurts L, Ouwerkerk JP, Druart C, Bindels LB (2013). Cross-talk between *Akkermansia muciniphila* and intestinal epithelium controls diet-induced obesity. Proc Natl Acad Sci U S A.

[112] Xu J, Gordon JI (2003). Honor thy symbionts. Proc Natl Acad Sci U S A.

[113] Stappenbeck TS, Hooper LV, Gordon JI (2002). Developmental regulation of intestinal angiogenesis by indigenous microbes via Paneth cells. Proc Natl Acad Sci U S A.

[114] Ivanov II, Atarashi K, Manel N, Brodie EL, Shima T, Karaoz U (2009). Induction of intestinal Th17 cells by segmented filamentous bacteria. Cell.

[115] Schnupf P, Gaboriau-Routhiau V, Cerf-Bensussan N (2013). Host interaction with segmented filamentous bacteria: an unusual trade-off that drives the post-natal maturation of the gut immune system. Semin Immunol.

[116] Chappert P, Bouladoux N, Naik S, Schwartz RH (2013). Specific gut commensal flora locally alters T cell tuning to endogenous ligands. Immunity.

[117] Kriegel MA, Sefik E, Hill JA, Wu HJ, Benoist C, Mathis D (2011). Naturally transmitted segmented filamentous bacteria segregate with diabetes protection in nonobese diabetic mice. Proc Natl Acad Sci U S A.

[118] Wu HJ, Ivanov II, Darce J, Hattori K, Shima T (2010). Gut-residing segmented filamentous bacteria drive autoimmune arthritis via T helper17 cells. Immunity.

[119] Pamp SJ, Harrington ED, Quake SR, Relman DA, Blainey PC (2012). Single-cell sequencing provides clues about the host interactions of segmented filamentous bacteria (SFB). Genome Res.

[120] Prakash T, Oshima K, Morita H, Fukuda S, Imaoka A (2011). Complete genome sequences of rat and mouse segmented filamentous bacteria, a potent inducer of th17 cell differentiation. Cell Host Microbe.

[121] Sczesnak A, Segata N, Qin X, Gevers D, Petrosino JF, Huttenhower C (2011). The genome of Th17 cell-inducing segmented filamentous bacteria reveals extensive auxotrophy and adaptations to the intestinal environment. Cell Host Microbe.

[122] Sano T, Huang W, Hall JA, Yang Y, Chen A, Gavzy SJ (2015). An IL-23R/IL-22 circuit regulates epithelial serum amyloid A to promote local effector Th17 responses. Cell.

[123] Atarashi K, Tanoue T, Ando M, Kamada N, Nagano K, Narushima S (2015). Th17 Cell induction by adhesion of microbes to intestinal epithelial cells. Cell.

[124] Maynard CL, Elson CO, Hatton RD, Weaver CT (2012). Reciprocal interactions of the intestinal microbiota and immune system. Nature.

[125] Neish AS, Gewirtz AT, Zeng H, Young AN, Hobert ME, Karmali V (2000). Prokaryotic regulation of epithelial responses by inhibition of IκB-α ubiquitination. Science.

[126] Kelly D, Campbell JI, King TP, Grant G, Jansson EA, Coutts AG (2004). Commensal anaerobic gut bacteria attenuate inflammation by regulating nuclear-cytoplasmic shuttling of PPAR-gamma and RelA. Nat Immunol.

[127] Tien MT, Girardin SE, Regnault B, Le Bourhis L, Dillies MA, Coppée JY (2006). Anti-inflammatory effect of *Lactobacillus casei* on Shigella-infected human intestinal epithelial cells. J Immunol.

[128] D’Inca R, Barollo M, Scarpa M, Grillo AR, Brun P, Vettorato MG (2011). Rectal administration of *Lactobacillus casei* DG modifies flora composition and toll-like receptor expression in colonic mucosa of patients with mild ulcerative colitis. Dig Dis Sci.

[129] Shen Y, Torchia MLG, Lawson GW, Karp CL, Ashwell JD, Mazmanian SK (2012). Outer membrane vesicles of a human commensal mediate immune regulation and disease protection. Cell Host Microbe.

[130] Bansal T, Alaniz RC, Wood TK, Jayaraman A (2010). The bacterial signal indole increases epithelial-cell tight-junction resistance and attenuates indicators of inflammation. Proc Natl Acad Sci U S A.

[131] Shimada Y, Kinoshita M, Harada K, Mizutani M, Masahata K, Kayama H (2013). Commensal bacteria-dependent indole production enhances epithelial barrier function in the colon. PLoS One.

[132] Chimerel C, Emery E, Summers DK, Keyser U, Gribble FM, Reimann F (2014). Bacterial metabolite indole modulates incretin secretion from intestinal enteroendocrine L cells. Cell Rep.

[133] Stamm I, Lottspeich F, Plaga W (2005). The pyruvate kinase of *Stigmatella aurantiaca* is an indole binding protein and essential for development. Mol Microbiol.

[134] Chant EL, Summers DK (2007). Indole signalling contributes to the stable maintenance of *Escherichia coli* multicopy plasmids. Mol Microbiol.

[135] Nikaido E, Yamaguchi A, Nishino K (2008). AcrAB multidrug efflux pump regulation in *Salmonella enterica* serovar Typhimurium by RamA in response to environmental signals. J Biol Chem.

[136] Lee J, Bansal T, Jayaraman A, Bentley WE, Wood TK (2007). Enterohemorrhagic *Escherichia coli* biofilms are inhibited by 7-hydroxyindole and stimulated by isatin. Appl Environ Microbiol.

[137] Lee J, Jayaraman A, Wood TK (2007). Indole is an inter-species biofilm signal mediated by SdiA. BMC Microbiol.

[138] Anyanful A, Dolan-Livengood JM, Lewis T, Sheth S, Dezalia MN, Sherman MA (2005). Paralysis and killing of *Caenorhabditis elegans* by enteropathogenic *Escherichia coli* requires the bacterial tryptophanase gene. Mol Microbiol.

[139] Scherer CA, Mille SI, Groisman EA (2001). Molecular pathogenesis of Salmonellae. Principles of bacterial pathogenesis.

[140] Nikaido E, Giraud E, Baucheron S, Yamasaki S, Wiedemann A, Okamoto K (2012). Effects of indole on drug resistance and virulence of *Salmonella enterica* serovar Typhimurium revealed by genome-wide analyses. Gut Pathog.

[141] Lee HH, Molla MN, Cantor CR, Collins JJ (2010). Bacterial charity work leads to population-wide resistance. Nature.

[142] Oh S, Go GW, Mylonakis E, Kim Y (2012). The bacterial signalling molecule indole attenuates the virulence of the fungal pathogen *Candida albicans*. J Appl Microbiol.

[143] Mueller RS, Beyhan S, Saini SG, Yildiz FH, Bartlett DH (2009). Indole acts as an extracellular cue regulating gene expression in *Vibrio cholera*. J Bacteriol.

[144] Venkatesh M, Mukherjee S, Wang H, Li H, Sun K, Benechet AP (2014). Symbiotic bacterial metabolites regulate gastrointestinal barrier function via the xenobiotic sensor PXR and toll-like receptor 4. Immunity.

[145] Lee J–H, Lee J (2010). Indole as an intercellular signal in microbial communities. FEMS Microbiol Rev.

[146] Delzenne NM, Neyrinck AM, Backhed F, Cani PD (2011). Targeting gut microbiota in obesity: effects of prebiotics and probiotics. Nat Rev Endocrinol.

[147] Claes A-K, Zhou JY, Philpot DJ (2015). NOD-l like receptors: guardians of intestinal mucosal barriers. Physiology.

[148] Jin C, Flavell RA (2013). Innate sensors of pathogen and stress: Linking inflammation to obesity. J Allergy Clin Immunol.

[149] Backhed F, Ding H, Wang T, Hooper LV, Koh GY, Nagy A (2004). The gut microbiota as an environmental factor that regulates fat storage. Proc Natl Acad Sci U S A.

[150] Backhed F, Manchester JK, Semenkovich CF, Gordon JI (2007). Mechanisms underlying the resistance to diet-induced obesity in germ-free mice. Proc Natl Acad Sci U S A.

[151] Dewulf EM, Cani PD, Neyrinck AM, Possemiers S, Van Holle A, Muccioli GG (2011). Inulin-type fructans with prebiotic properties counteract GPR43 overexpression and PPARγ-related adipogenesis in the white adipose tissue of high-fat diet-fed mice. J Nutr Biochem.

[152] Turnbaugh PJ, Ley RE, Mahowald MA, Magrini V, Mardis ER, Gordon JI (2006). An obesity-associated gut microbiome with increased capacity for energy harvest. Nature.

[153] Spor A, Koren O, Ley R (2011). Unravelling the effects of the environment and host genotype on the gut microbiome. Nat Rev Microbiol.

[154] Lopetuso LR, Scaldaferri F, Bruno G, Petito V, Franceschi F, Gasbarrini A (2015). The therapeutic management of gut barrier leaking: the emerging role for mucosal barrier protectors. Eur Rev Med Pharmacol Sci.

[155] Hsiao WWL, Metz C, Singh DP, Roth J (2008). The microbes of the intestine: an introduction to their metabolic and signaling capabilities. Endocrinol Metab Clin N Am.

[156] Boerner BP, Sarvetnick NE (2011). Type 1 diabetes: role of intestinal microbiome in humans and mice. Ann N Y Acad Sci.

[157] Hara N, Alkanani AK, Ir D, Robertson CE, Wagner BD, Frank DN (2013). The role of the intestinal microbiota in type 1 diabetes. Clin Immunol.

[158] Wen L, Ley RE, Volchkov PY, Stranges PB, Avanesyan L, Stonebraker AC (2008). Innate immunity and intestinal microbiota in the development of Type 1 diabetes. Nature.

[159] McInerney MF, Pek SB, Thomas DW (1991). Prevention of insulitis and diabetes onset by treatment with complete Freund’s adjuvant in NOD mice. Diabetes.

[160] Hansen CHF, Krych L, Nielsen DS, Vogensen FK, Hansen LH, Sørensen SJ (2012). Early life treatment with vancomycin propagates *Akkermansia muciniphila* and reduces diabetes incidence in the NOD mouse. Diabetologia.

[161] Amar J, Chabo C, Waget A, Klopp P, Vachoux C, Bermúdez-Humarán LG (2011). Intestinal mucosal adherence and translocation of commensal bacteria at the early onset of type 2 diabetes: molecular mechanisms and probiotic treatment. EMBO Mol Med.

[162] Toivonen RK, Emani R, Munukka E, Rintala A, Laiho A, Pietilä S (2014). Fermentable fibres condition colon microbiota and promote diabetogenesis in NOD mice. Diabetologia.

[163] Wright EK, Kamm MA, Teo SM, Inouye M, Wagner J, Kirkwood CD (2015). Recent advances in characterizing the gastrointestinal microbiome in Crohn’s disease: A systematic review. Inflamm Bowel Dis.

[164] Knip M, Siljander H (2016). The role of the intestinal microbiota in type 1 diabetes mellitus. Nat Rev Endocrinol.

[165] Cesaro C, Tiso A, Del Prete A, Cariello R, Tuccillo C, Cotticelli G (2011). Gut microbiota and probiotics in chronic liver diseases. Dig Liver Dis.

[166] Hartmann P, Haimerl M, Mazagova M, Brenner DA, Schnabl B (2012). Toll-like receptor 2-mediated intestinal injury and enteric tumor necrosis factor receptor I contribute to liver fibrosis in mice. Gastroenterology.

[167] Mazagova M, Wang L, Anfora AT, Wissmueller M, Lesley SA, Miyamoto Y (2015). Commensal microbiota is hepatoprotective and prevents liver fibrosis in mice. FASEB J.

[168] Henao-Mejia J, Elinav E, Jin C, Hao L, Mehal WZ, Strowig T (2012). Inflammasome-mediated dysbiosis regulates progression of NAFLD and obesity. Nature.

[169] Henao-Mejia J, Elinav E, Thaiss CA, Licona-Limon P, Flavell RA (2013). Role of the intestinal microbiome in liver disease. J Autoimmun.

[170] He C, Shan Y, Song W (2015). Targeting gut microbiota as a possible therapy for diabetes. Nutr Res.

[171] Saponaro C, Gaggini M, Gastaldelli A (2015). Nonalcoholic fatty liver disease and type 2 diabetes: common pathophysiologic mechanisms. Curr Diab Rep.

[172] Couvigny B, de Wouters T, Kaci G, Jacouton E, Delorme C, Doré J (2015). Commensal *Streptococcus salivarius* modulates PPARγ transcriptional activity in human intestinal epithelial cells. PLoS One.

[173] Korecka A, de Wouters T, Cultrone A, Lapaque N, Pettersson S, Dore J (2013). ANGPTL4 expression induced by butyrate and rosiglitazone in human intestinal epithelial cells utilizes independent pathways. Am J Physiol Gastrointest Liver Physiol.

[174] Kaci G, Goudercourt D, Dennin V, Pot B, Dore J, Ehrlich SD (2014). Anti-inflammatory properties of *Streptococcus salivarius,* a commensal bacterium of the oral cavity and digestive tract. Appl Environ Microbiol.

[175] Ochoa-Repáraz J, Mielcarz DW, Begum-Haque S, Kasper LH (2011). Gut, bugs, and brain: role of commensal bacteria in the control of central nervous system disease. Ann Neurol.

[176] Lyte M, Lyte M, Cryan JF (2014). Microbial endocrinology and the microbiota-gut-brain axis. Microbial endocrinology: The microbiota gut-brain axis in health and disease.

[177] Lyte M (1993). The role of microbial endocrinology in infectious disease. J Endocrinol.

[178] Lyte M, Lyte M, Freestone PPE (2010). Microbial endocrinology: a personal journey. Microbial endocrinology: interkingdom signaling in infectious disease and health.

[179] Barbara G, Stanghellini V, De GR, Cremon C, Cottrell GS, Santini D (2004). Activated mast cells in proximity to colonic nerves correlate with abdominal pain in irritable bowel syndrome. Gastroenterology.

[180] Bravo JA, Forsythe P, Chew MV, Escaravage E, Savignac HM, Dinan TG, Bienenstock J, Cryan JF (2011). Ingestion of Lactobacillus strain regulates emotional behavior and central GABA receptor expression in a mouse via the vagus nerve. Proc Natl Acad Sci U S A.

[181] Li W, Dowd S, Scurlock B, Acosta-Martinez V, Lyte M (2009). Memory and learning behavior in mice is temporally associated with diet-induced alterations in gut bacteria. Physiol Behav.

[182] Grenham S, Clarke G, Cryan JF, Dinan TG (2011). Brain-gut-microbe communication in health and disease. Front Physiol.

[183] Blanchard EB, Scharff L, Schwarz SP, Suls JM, Barlow DH (1990). The role of anxiety and depression in the irritable bowel syndrome. Behav Res Ther.

[184] Erny D, de Angelis ALH, Jaitin D, Wieghofer P, Staszewski O, David E (2015). Host microbiota constantly control maturation and function of microglia in the CNS. Nat Neurosci.

[185] Prinz M, Priller J (2014). Microglia and brain macrophages in the molecular age: from origin to neuropsychiatric disease. Nat Rev Neurosci.

[186] Schafer DP, Stevens B (2013). Phagocytic glial cells: sculpting synaptic circuits in the developing nervous system. Curr Opin Neurobiol.

[187] Jacobs JP, Braun J (2014). Immune and genetic gardening of the intestinal microbiome. FEBS Lett.

[188] Petnicki-Ocwieja T, Hrncir T, Liu Y-J, Biswas A, Hudcovic T, Tlaskalova-Hogenova H (2009). Nod2 is required for the regulation of commensal microbiota in the intestine. Proc Natl Acad Sci U S A.

[189] Rehman A, Sina C, Gavrilova O, Häsler R, Ott S, Baines JF (2011). Nod2 is essential for temporal development of intestinal microbial communities. Gut.

[190] Vijay-Kumar M, Aitken JD, Carvalho FA, Cullender TC, Mwangi S, Srinivasan S (2010). Metabolic syndrome and altered gut microbiota in mice lacking Toll-like receptor 5. Science.

[191] Larsson E, Tremaroli V, Lee YS, Koren O, Nookaew I, Fricker A (2012). Analysis of gut microbial regulation of host gene expression along the length of the gut and regulation of gut microbial ecology through MyD88. Gut.

[192] Sokol H, Pigneur B, Watterlot L, Lakhdari O, Bermúdez-Humarán LG, Gratadoux JJ (2008). *Faecalibacterium prausnitzii* is an anti-inflammatory commensal bacterium identified by gut microbiota analysis of crohn disease patients. Proc Natl Acad Sci U S A.

[193] Foligne B, Nutten S, Grangette C, Dennin V, Goudercourt D, Poiret S (2007). Correlation between in vitro and in vivo immunomodulatory properties of lactic acid bacteria. World J Gastroenterol.

[194] Veazey RS, Lackner AA (2005). HIV swiftly guts the immune system. Nat Med.

[195] Sandler NG, Douek DC (2012). Microbial translocation in HIV infection: causes, consequences and treatment opportunities. Nat Rev Microbiol.

[196] Vujkovic-Cvijin I, Dunham RM, Iwai S, Maher MC, Albright RG, Broadhurst MJ (2013). Dysbiosis of the gut microbiota is associated with HIV disease progression and tryptophan catabolism. Sci Transl Med.

[197] Lozupone CA, Li M, Campbell TB, Flores SC, Linderman D, Gebert MJ (2013). Alterations in the gut microbiota associated with HIV-1 infection. Cell Host Microbe.

[198] McHardy IH, Li X, Tong M, Ruegger P, Jacobs J, Borneman J (2013). HIV Infection is associated with compositional and functional shifts in the rectal mucosal microbiota. Microbiome.

[199] Diaz PI, Hong BY, Frias-Lopez J, Dupuy AK, Angeloni M, Abusleme L (2013). Transplantation-associated long-term immunosuppression promotes oral colonization by potentially opportunistic pathogens without impacting other members of the salivary bacteriome. Clin Vaccine Immunol.

[200] Round JL, Lee SM, Li J, Tran G, Jabri B, Chatila TA (2011). The Toll-like receptor 2 pathway establishes colonization by a commensal of the human microbiota. Science.

[201] Goodwin AC, Destefano Shields CE, Wu S, Huso DL, Wu X, Murray-Stewart TR (2011). Polyamine catabolism contributes to enterotoxigenic *Bacteroides fragilis*-induced colon tumorigenesis. Proc Natl Acad Sci U S A.

[202] Garrett WS (2015). Cancer and the microbiota. Science.

[203] Pamer EG (2007). Immune responses to commensal and environmental microbes. Nat Immunol.

[204] Qin J, Li Y, Cai Z, Li S, Zhu J, Zhang F (2012). A metagenome-wide association study of gut microbiota in type 2 diabetes. Nature.

[205] Le Roy T, Llopis M, Lepage P, Bruneau A, Rabot S, Bevilacqua C (2013). Intestinal microbiota determines development of non-alcoholic fatty liver disease in mice. Gut.

[206] Li D, Zhang L, Dong F, Liu Y, Li N, Li H (2015). Metabonomic changes associated with atherosclerosis progression for LDLR−/− mice. J Proteome Res.

